# Online capacitance-integrated development of a hybrid bioprocess model for industrial fungal fermentations

**DOI:** 10.3389/fbioe.2026.1919748

**Published:** 2026-10-05

**Authors:** Marc Lemperle, Julian Kager, Benny Cassells, Stuart M. Stocks, Krist V. Gernaey, Pedram Ramin

**Affiliations:** 1 Process and Systems Engineering Center (PROSYS), Department of Chemical and Biochemical Engineering, Technical University of Denmark, Kongens Lyngby, Denmark; 2 Novonesis, Fermentation Pilot Plant, Lyngby, Denmark; 3 Novo Nordisk Foundation Biotechnology Research Institute for the Green Transition (BRIGHT), Technical University of Denmark, Lyngby, Denmark

**Keywords:** bioprocess model, capacitance data, experimental design, hybrid modelling, industrial fungal fermentati

## Abstract

The growing demand for fermentation-based products has increased interest in bioprocess modelling approaches compatible with routinely available industrial process data. Capacitance probes are promising because they provide online data related to viable biomass, but their use in filamentous fungal fermentations remains challenging, as state-of-the-art cell-count-based calibration methods of capacitance data are not applicable to hyphal organisms under industrial settings. This study presents a capacitance-integrated hybrid modelling workflow for highly viscous, enzyme-producing filamentous fungal fermentations at pilot scale under data-scarce conditions. The oxygen mass transfer coefficient $k_{L}a$ was developed using an intra-batch design covering agitation, pressure, and aeration dynamics. The correlation achieved a model fit of $R^{2}=0.95$ and approximately 8% relative validation error. Capacitance data were used to define a pseudo-viable biomass state based on a single CDW-derived anchor point, reducing the need for repeated offline CDW measurements. A ±20% perturbation of this calibration point had only a minor effect on overall model accuracy within the dataset investigated. The hybrid model was assessed on two pilot-scale fermentations with distinct operating trajectories. One of the two batches was used for viscosity model selection and therefore does not represent a fully independent validation batch. Product concentration was predicted with NRMSE values of 5.8% for a closed-loop and 4.3% for an open-loop simulation, although several flow-, oxygen-transfer-, and biomass-related states showed larger deviations. Overall, the results provide a proof of concept for capacitance-integrated hybrid modelling, reducing the number of fermentation batches by 66.7% and decreasing offline sampling requirements from previously 10–15 samples for both, CDW and viscosity per batch to one CDW sample and no viscosity samples.

## Graphical Abstract

**Figure:**
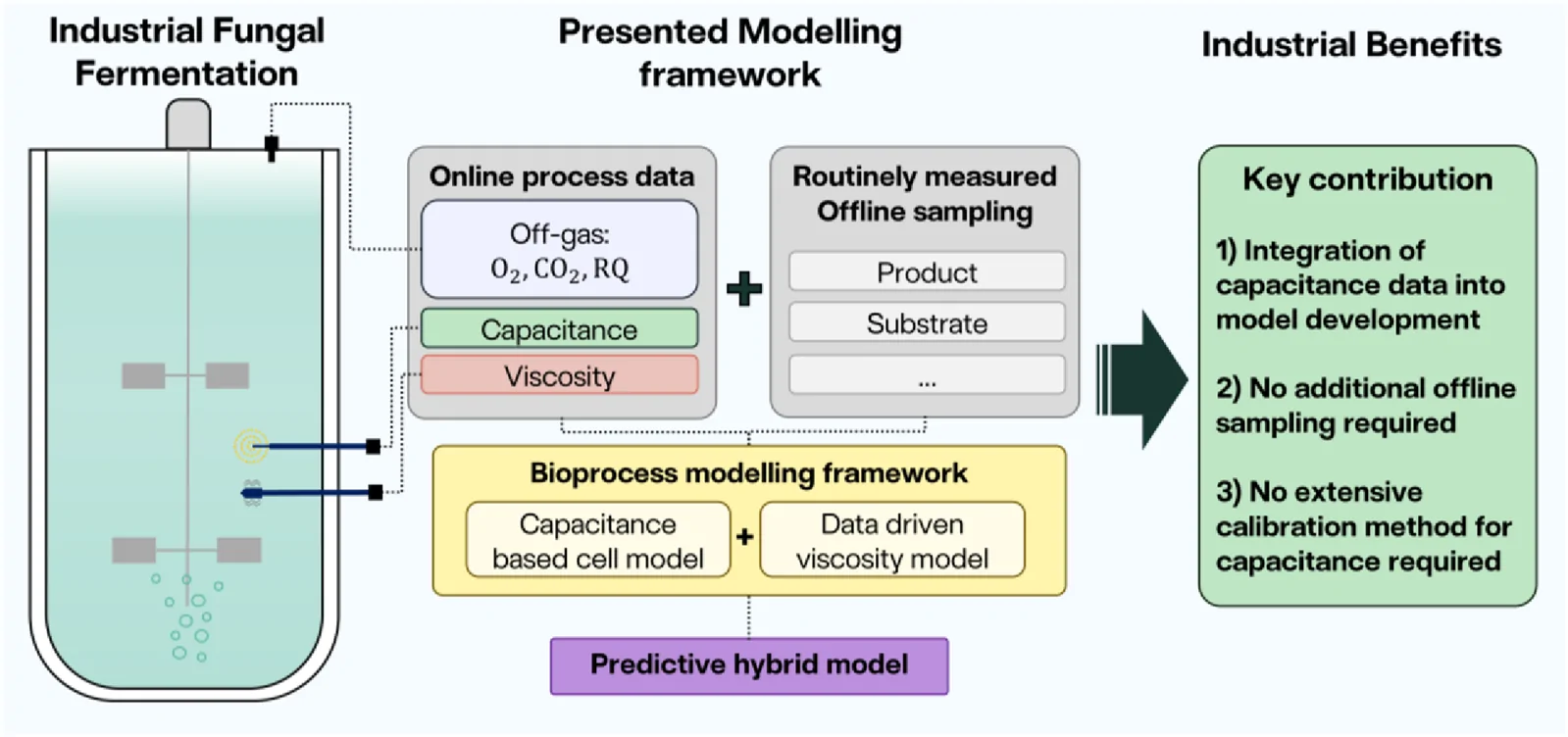

## Introduction

The biotechnological manufacturing industry constantly faces major challenges in meeting the growing demand for biological products such as pharmaceuticals or industrial enzymes (Pandey et al., 2023; Pörtner, 2023). This growth in demand requires advances in production efficiency, quality control and process scalability (Kantaros et al., 2025; Pandey et al., 2023; Shahab et al., 2025).

In recent years, the modelling of chemical and biochemical processes has attracted considerable attention, particularly within process systems engineering. Models have long served as fundamental tools for improving process understanding and control, design and optimization. However, the development of such digital representations of physical and biological systems still relies heavily on manual, labor-intensive data generation and process characterization. Model development requires a rigorous investigation of the fundamental principles governing the process and is typically accompanied by extensive experimental work. Consequently, the development of accurate and functional mathematical models is resource-intensive and time-consuming, resulting in limited implementation in highly complex industrial environments (Heyer et al., 2025; Laub et al., 2025). Many critical process variables such as substrate concentration and biomass are still monitored in an offline manner across the industry (Ferreira et al., 2005).

Traditionally, offline measurements such as optical density (OD) and cell dry weight (CDW) are used to estimate biomass concentration. However, these methods have well-known limitations in many industrial bioprocesses, particularly when complex media components are used (Bergin et al., 2022). Such limitations are also highly relevant in industrial fungal fermentation for enzyme production, where insoluble material precipitated medium components and accumulated cell debris can interfere with CDW and OD measurements. Moreover, CDW analysis involves time-consuming sampling, washing, drying, and weighing steps, which restricts its use for timely process monitoring and control (Magnússon et al., 2023; Rønnest et al., 2011).

Capacitance probes offer a promising alternative, as they enable real-time online monitoring of a variety of cell-specific traits, such as viable cell volume, cell size or cell culture homogeneity (Ehgartner et al., 2017). Although capacitance probes are increasingly established as monitoring tools in mammalian cell culture processes, several challenges remain regarding the use of their signal outputs for model-based applications (Metze et al., 2019; Surowiec and Scholz, 2023).

First, their capacitance measurements are typically reported in non-standard units such as picofarads/cm^2^, which complicates direct correlation with conventional biomass metrics, for example, grams of viable cells per kilogram of fermentation broth. Calibration approaches are traditionally based on cell staining or cell counting methods (e.g., CFUs, colony-forming units), which are either infeasible for fungal strains or would introduce additional, non-routine analytical steps into the modelling framework. This limitation arises from the filamentous growth of fungi, growing as branched hyphal networks rather than as individual cells. Since capacitance primarily reflects the total volume of viable, membrane-enclosed biomass, the capacitance does not directly correlate with cell number in filamentous fungal fermentations (Magnússon et al., 2023). Numerous studies have explored correlations between CDW and capacitance measurements, although CDW inherently represents the total non-soluble solids in the medium, not exclusively cells, and particularly not viable cells (Dabros et al., 2009; Ehgartner et al., 2015; Konakovsky et al., 2015). While previous studies have demonstrated capacitance-based monitoring and soft-sensor approaches in bioprocesses, fewer studies have addressed their integration into modelling industrial filamentous fungal fermentations, where cell-count-based calibration is impractical. This study therefore investigates whether an online capacitance-derived pseudo-viable biomass can be used as a biomass approximation for a hybrid model of an industrially relevant fermentation process at pilot scale (Downey et al., 2013; Kyriakopoulos et al., 2018; Lemke et al., 2023; Metze et al., 2019; Rafferty et al., 2020).

In filamentous fungal fermentations, the increase in biomass is closely linked to the broth rheology, as hyphal elongation or branching may lead to increased viscosity and non-Newtonian behavior (Olsvik and Kristiansen, 1994; Roels et al., 1974; Villadsen et al., 2011). As a result, viscosity has long been recognized as another key process parameter reflecting both fungal morphology and biomass development (García-Ochoa and Gómez, 1998). Similar to offline CDW and OD measurements for biomass estimation, offline rheological measurements used to determine apparent viscosity are associated with several limitations. They are often error-prone, affected by sample handling, and dependent on assumptions regarding shear conditions and sample representativeness. In contrast, online viscosity probes (i.e., vibrational viscometer) are known for their ability to characterize the rheology of fluids in real time, offering significant potential for model development (Lemperle et al., 2026; Myer, 2015; Pu et al., 2015). The transition from traditional offline rheology measurements, typically used to approximate apparent viscosity, to online monitoring helps to streamline modelling and simplify the complexity of rheological data collection and interpretation (Allen and Robinson, 1990; Sun et al., 2024; Wucherpfennig et al., 2010).

In parallel, the process modelling community is increasingly adopting data-driven approaches to develop more cost-effective and accurate models. Because the performance of machine learning methods in bioprocessing strongly depends on data availability and quality, new modelling strategies have emerged that are either fully data-driven or combine mechanistic and data-driven components, commonly referred to as hybrid models (Bradley et al., 2022; Kadlec et al., 2009; Oliveira, 2004; von Stosch et al., 2014).

In summary, online process data is increasingly used in the monitoring of bioprocesses and model development to reduce reliance on frequent offline sampling. In particular, off-gas measurements, such as oxygen uptake rate, carbon dioxide evolution rate, and respiratory quotient, are well-established tools for describing process physiology and supporting model calibration (Albaek et al., 2011). However, the use of online capacitance-based biomass for model development for industrial filamentous fungal fermentations remains less explored.

Therefore, this study aims to narrow the gap between traditional, manual, time- and resource-intensive model development approaches and a workflow that further reduces reliance on offline sampling, particularly cell dry weight (CDW). We emphasize the utilization of online data, including online biomass and online viscosity measurements, while reducing offline measurements to a bare minimum. Offline data is limited to a high degree to routinely measured process variables, such as product or substrate concentrations, which are typically monitored in industry and pose no additional workload (Nadal-Rey, 2020). Within this scope, we investigate whether complex calibration procedures for capacitance probe data can be avoided by using a single CDW-derived calibration anchor to define a pseudo-viable biomass state. Furthermore, we present industrial data from four pilot-scale fermentations, focusing on five distinct capacitance probe measurements and evaluating their suitability for modelling purposes in a pre-modelling step. The workflow was evaluated using two pilot-scale batches with distinct operating conditions, including validation on one batch and further assessment under different operating conditions on the other. The resulting model performance is interpreted as proof-of-concept evidence.

## Materials and methods

### Experimental data

The four fermentation runs, as described in Table 1, were designed with the objective of generating a dataset that supports a modelling framework requiring as little offline sampling as possible beyond what is routinely measured in industrial fermentation. All experiments were performed at the pilot plant of Novonesis A/S, Bagsværd, using 550L STR reactors (Bach et al., 2017). Fermentation 1 is used to generate a comprehensive dataset for fitting an empirical $k_{L}a$ correlation. To achieve this, pressure, agitation, and aeration were systematically varied in a continuous pattern and across the operational space to maximize coverage of $k_{L}a$ values. This simultaneously ensured that all corner points, as well as intermediate points within the design space, were considered. This method is further developed from the intra-batch calibration of $k_{L}a$ , presented in Lemperle et al. (2026).

**TABLE 1:** Overview of four fermentation runs used for establishing a capacitance integrated development of the hybrid bioprocess model.

Fermentations	Name	Objective	Characteristics	Control	Model development application
1	Dynamic Pressure–Agitation–Aeration (PAA) Profile	Investigate $k_{L}a$ correlation + Investigates biology of three different growth rates	Dynamic setpoints for pressure agitation and aeration	Manipulated variable: feed rate Controlled variable: dissolved oxygen	Used for $k_{L}a$ model, viscosity ML and biological model
2	Fixed Feed–Targeted Growth	Investigates biology of three different growth rates	Three different feeding profiles, each targeting an individual growth rate	Open-loop, pre-designed feed rate	Used for viscosity, ML and biological model
3	Inverse PAA Profile	$k_{L}a$ model validation and selection of the data-driven viscosity model	Reverse order of the same pattern used in Fermentation 1	Manipulated variable: feed rate Controlled variable: dissolved oxygen	$k_{L}a$ model validation and selection of the data-driven viscosity model
4	Open loop, low OTR setting	Hybrid model validation	Lowest DoE settings of aeration, pressure, and agitation	Open-loop, pre-designed feed rate	Hybrid model validation

In addition, Fermentation 2 further investigates the relationship between growth rates and intracellular dynamics by correlating three distinct growth rates with cell-specific yields ( $Y_{SX_{v}}$ , $Y_{O_{2}X_{v}},Y_{CO_{2}X_{v}}$ and $Y_{{\text{NH}}_{4}^{+}X_{v}}$ ). Feed rates were predetermined based on the estimated cell-specific substrate uptake rate $\frac{Xv}{Y_{SX}}*W$ and targeted growth rates μ , as presented in Equation 1. Aeration, pressure, and agitation conditions were designed to prevent oxygen depletion. As such, this approach ensured that dissolved oxygen remained above 15% throughout the process.

$$F_{in}=\mu *\;\frac{X}{Y_{SX_{v}}*c_{F}}*W$$ (1)

Fermentation 3 was designed to reproduce the agitation, pressure, and aeration dynamics of Fermentation 1, but in reverse order, thereby providing an independent batch with similar operating-space coverage but a different temporal sequence. It was used to evaluate whether the $k_{L}a$ model, fitted with the data from Fermentation 1, could reproduce the process response under altered dynamic trajectories of agitation, pressure and aeration. In addition, Fermentation 3 was used to validate and select the data-driven viscosity model later utilized in the hybrid model.

Fermentation 4 was run with the lowest DoE set points of aeration, pressure and agitation and without DO-control to increase the dataset variability and to validate the final hybrid model.

In a pre-model development step, the data from all 4 fermentation runs were used to evaluate the suitability of each capacitance measurement for modelling biomass.

Since fermentation 3 was used during sub-model selection for the hybrid model, including the data-driven viscosity model, the reported final performance metrics should be interpreted as optimistic estimates rather than as fully independent external validation. Only fermentation 4 was fully held out for independent validation for the hybrid model.

### Capacitance probe measurements

A capacitance probe measures the capacitance (i.e., linked to permittivity, which equals the capacitance [F] per geometry cell constant K $\left[\frac{1}{m}\right]$ ) in a defined volume of liquid over a frequency range generated by an applied electric field. The resulting data may be presented as a β-dispersion curve, which represents permittivity, interpretable as the ability of the cells, present within the measurement volume to store electrical charge, as a function of frequency. Figure 1 visualizes a β-dispersion curve, which provides several insights into cell-specific properties. First, it indicates the degree to which the cell volume is polarized, providing information about the total viable cell volume. In this work, the capacitance-derived viable biovolume signal is referred to as apparent viable cell concentration for simplicity. This term should be understood as a capacitance-based proxy for viable biomass or viable cell volume per culture volume, rather than as a direct cell count concentration. In addition, the frequency-dependent capacitance response is influenced by cell size and morphological characteristics, providing complementary information on cell-specific properties. The viable cell concentration can be interpreted using three different dual-frequency approaches. The first and second approach, referred to as microbial mode and bacterial mode, defined as in Equations 2, 3, respectively quantify viable biomass by calculating the difference between the permittivity measured at a cell-type-specific frequency $\left(microbial:f_{m}=580\;kHz,bacterial:f_{b}=779\;kHz\right)$ and the permittivity measured at a sufficiently high frequency $\left(=15\;650\;\text{kHz}\right)$ , which serves as a background reference.

$$\Delta \varepsilon_{\text{microbial}}=\varepsilon \left(f_{m}\right)-\varepsilon \left(f_{\text{HF}}\right)$$ (2)

$$\Delta \varepsilon_{\text{bacterial}}=\varepsilon \left(f_{b}\right)-\varepsilon \left(f_{\text{HF}}\right)$$ (3)

where $\varepsilon \left(f_{\text{HF}}\right)$ represents the high-frequency reference signal that is largely insensitive to viable biomass. According to the manufacturer’s recommendations, the microbial mode is suitable for filamentous fungi. Therefore, no separate fungal-specific mode was investigated in this study.

**FIGURE 1:**
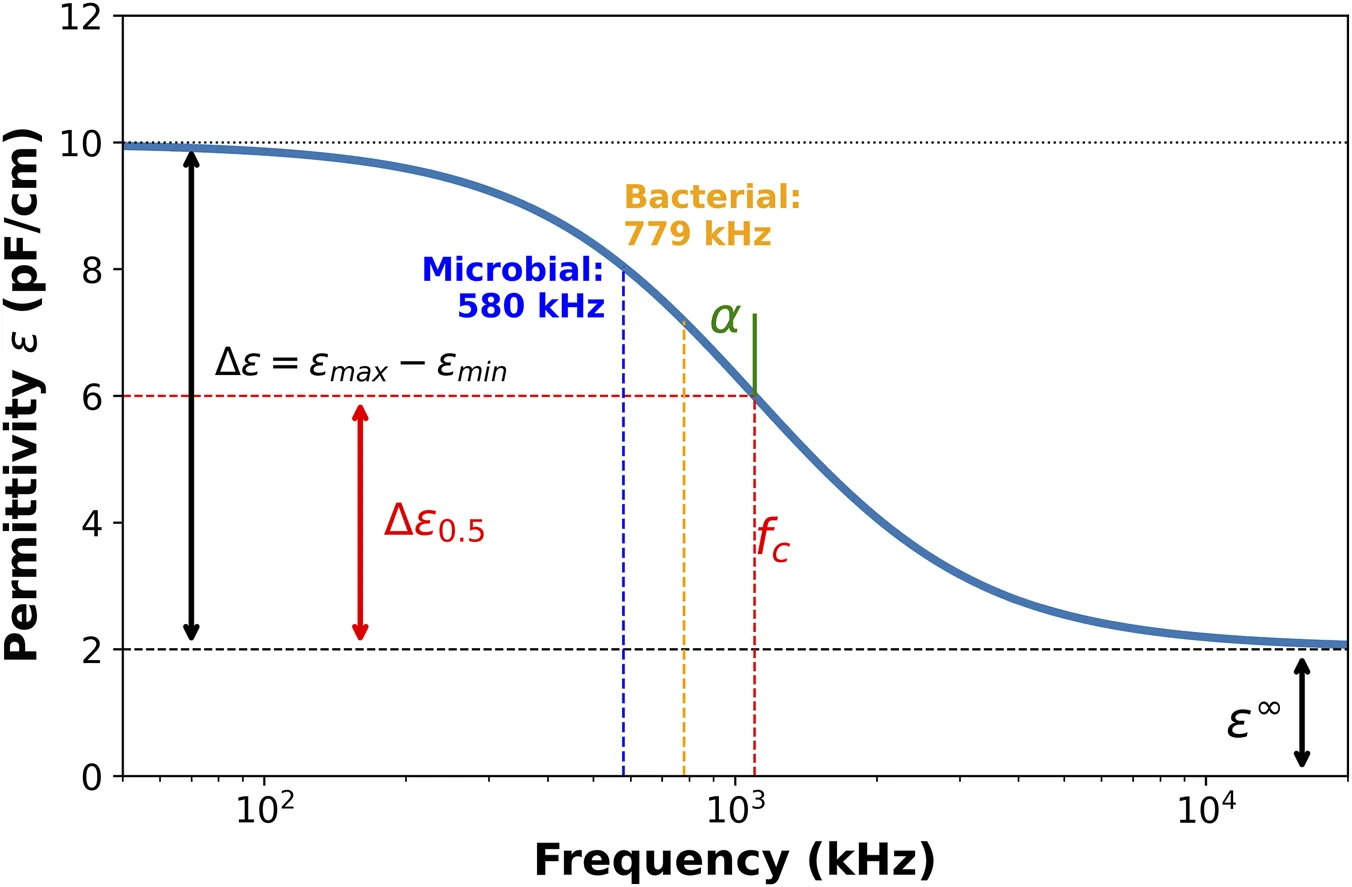
Representation of the beta-dispersion curve, based on the Cole-Cole model (Cole and Cole, 1941). It visualizes the key parameters such as delta Epsilon, the slope at the critical frequency, non-biological background interference ( $C^{\infty }$ ) and two additional dual frequency modes. The data presented in this study is recorded by an Aber Futura capacitance probe.

The third approach is based on the β-dispersion behavior of the permittivity signal and considers the difference between the maximum and minimum permittivity values of the β-dispersion curve, commonly referred to as Δε , as defined in Equation 4. $\varepsilon_{\max}$ and $\varepsilon_{\min}$ are not fixed and can vary according to the measurement.

$$\Delta \varepsilon =\varepsilon_{\max}-\varepsilon_{\min}$$ (4)

These three different measurement methods will be evaluated in terms of their suitability for modelling viable cells under different process conditions, prior to the modelling workflow.

In addition, the critical frequency ( $f_{c}$ ) serves as an indicator of cell size, with lower values corresponding to larger cells and higher values corresponding to smaller cells. The Cole-Cole alpha (α) parameter provides information about the heterogeneity of the population: Lower α values indicate a more homogeneous culture with a narrower relaxation time distribution, while higher α values reflect greater variability, which is often associated with broader relaxation time and cell size distributions (Konakovsky et al., 2015; Markx et al., 1991; Surowiec and Scholz, 2023).

### Calibration methods

The calibration method aims to relate one of the three capacitance measurements (ΔCap) to the product of cell dry weight (CDW), a viable fraction $v_{f,i}$ , and a calibration factor m , following the model, shown in Equation 5, proposed by Magnússon et al. (2023). The viable fraction is defined as the portion of the CDW sample, that consists of viable cells.

$$\Delta Cap=m\;*{v_{f}}_{i}*CDW_{i}$$ (5)

Although enforcing a perfectly linear relationship may seem to simplify the estimation of $v_{f,i}$ and m , it leads to an ill-posed problem, as the two parameters cannot be uniquely identified without independent measurements of the viable fraction.

To overcome this, we assume that there exists a time point during the fermentation, presumably during the late exponential growth phase, where the viable fraction approaches 1 ( $v_{f}\approx 1$ ).

Under this assumption, the model simplifies to $m=\frac{\Delta Cap}{CDW}$ . In an industrial fungal fermentation process involving a seed transfer, the initial cell dry weight (CDW) measurements might be biased due to the presence of non-cellular and non-soluble particles introduced with the seed or in the starting batch media. At later stages of the fermentation, CDW measurements may again be distorted, this time due to the accumulation and precipitation of cell debris, residues or chemicals.

It is therefore reasonable to assume that the viable fraction of the CDW approaches 1 at a specific point during the exponential growth phase, particularly towards its later stages. The calibration factor m is therefore estimated using the maximum observed value of the specific capacitance ratio $\left(\frac{\Delta Cap\;}{CDW\;}\right)$ within the selected phase. This corresponds to a one-point calibration, summarized in Table 2. Since for a given strain, $\frac{\Delta Cap}{CDW}$ is assumed to be consistent, the one identified calibration factor is consistently used for the entire data set.

**TABLE 2:** Determination of the calibration factor m and viable fraction $v_{f}$ .

Variables	Objective	bounds
m *:* calibration factor	1-point calibration: $m=\max\left(\frac{\Delta Cap\;}{CDW\;}\right)$ , during exponential phase, assuming ${v_{f}}_{i}=1$	m>0
${v_{f}}_{i}:$ *Living fraction for each data point* i *(0 ≤* $vf_{i}$ *≤ 1)*	$v_{f_{i}}=\frac{\Delta CAP_{i}}{m*CDW_{i}}$ (6)	${v_{f}}_{i}\in \left[0,1\right]$

The calibrated value of m is subsequently used to estimate the viable fraction of the CDW measurements over time, as seen in Equation 6. (Justice et al., 2011; Raue et al., 2009). The viable fraction $v_{f_{i}}$ is not an independently measured viability value. It is derived from the capacitance-to-CDW calibration and is therefore partly imposed by the assumption that the maximum specific capacitance ratio corresponds to $v_{f_{i}}=1$ . Accordingly, $v_{f_{i}}$ is used here as an approximate indicator for interpreting biomass viability in relation to the CDW measurements, rather than as an independently validated viability measurement.

It is important to highlight that, under these assumptions, the resulting correlation does not provide the actual viable cell concentration, but rather an approximate *pseudo-viable cell concentration*.

It will be highlighted that this pseudo viable cell concentration is sufficient to build a full bioprocess model, including a mechanistic growth and death model with parameters in a biologically acceptable range.

### Three-point robustness analysis with respect to the calibration factor m

To assess the final error robustness of the simulated state predictions to uncertainty in the 1-point calibration factor m , a three-point robustness analysis was performed. The reference value of m was obtained from the one-point CDW-anchored calibration method. To mimic uncertainty associated with the calibration, two additional values were generated by perturbing the reference value by ±20%, resulting in $0.8\;m_{\text{ref}}$ , $m_{\text{ref}}$ , and $1.2\;m_{\text{ref}}$ (Marino et al., 2008).

For each value of m , the capacitance signal was converted into a pseudo-viable cell concentration, and the complete modelling workflow was repeated. The resulting state predictions were compared based on the NRMSE. This analysis provides a coarse empirical profile of the prediction error with respect to the calibration factor m and evaluates whether uncertainty in m affects the predicted process states, following the application of the hybrid modelling workflow. It is therefore used to assess the robustness of the modelling framework to changes in the calibration factor. The analysis is conceptually related to profile likelihood analysis, in the sense that the effect of fixing a parameter at different values is evaluated after repeating the model fitting workflow. However, instead of constructing a full likelihood profile over a dense range of m values, the present analysis uses three representative calibration scenarios and compares their effect on state prediction accuracy (Raue et al., 2009).

### Hybrid bioprocess modelling workflow

The modelling workflow, designed to limit the need for extraordinary and assumption heavy offline sampling, in particular, CDW and viscosity, is visualized in Figure 2.

**FIGURE 2:**
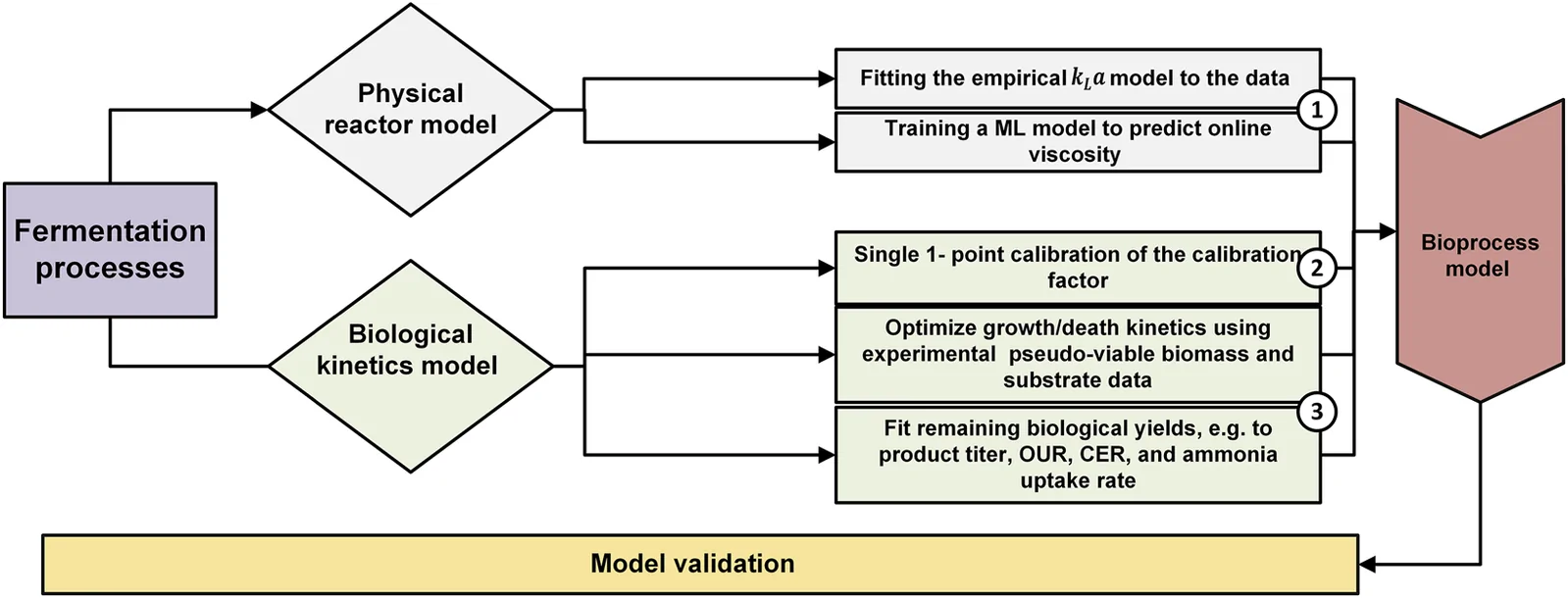
Bioprocess model workflow for industrial fungal fermentation. Two fermentation runs are used to generate data, which is subsequently used for the training and fitting of the physical and biological sub-models. Additional evaluation and validation were done with two additional runs.

To enable bioprocess model development for industrial fermentation processes while reducing the need for extraordinary offline sampling, this study proposes a workflow that leverages online viscosity (also referred to as dynamic or bulk viscosity) and online capacitance biomass measurements. The first part of the workflow focuses on the development of the physical reactor model, illustrated by the grey background in Figure 2. In this work, the physical reactor model includes a hybrid $k_{L}a$ model, which is described for the first time in Lemperle et al. (2026), and further developed in this work. The hybrid $k_{L}a$ model takes the following form, given in Equation 7:

$${\;k}_{L}a=C\;*{\left(\frac{P_{total}}{V}\right)}^{a}*v_{g}^{b}*{\left(f_{ML}\left(Y_{i}|i=1,\ldots n\right)\right)}^{c}$$ (7)

Here, $\frac{P_{total}}{V}$ denotes the specific power input (power per unit liquid volume), $v_{g}$ denotes the superficial gas velocity, and $Y_{i}$ represents the set of input process variables used by the machine-learning function $f_{ML}$ , where i=1,…,n .

The second and third parts focus on the biological kinetic model, highlighted by the green background in Figure 2. The second step determines the calibration factor, m , which scales the capacitance signal to obtain a pseudo viable cell concentration. In the present workflow, m is determined from a single CDW measurement, which serves as a one-time calibration anchor relating the capacitance-derived signal to offline biomass data. Once determined, here specifically with Fermentation 2, this calibration factor is kept constant and applied throughout the datasets and thus models across the investigated operating conditions.

Subsequently, a pool of growth and death models is evaluated through parameter optimization using data from Fermentation 1 and 2. This iterative procedure identifies the combination of models that are able to capture the cellular dynamics across the two markedly different fermentation runs. Since this selection is based on only two training fermentation runs and the data provide no independent measurement of dead biomass, the resulting growth and death models should be interpreted as candidate models rather than as uniquely identifiable biological models. In addition, a closed mass balance on total cell material would require explicit consideration of both viable and dead cell fractions. Since no dead-cell measurements were available, the corresponding dynamics were not modelled explicitly in this study.

Table 3 lists the set of growth and death models selected for this study that are iteratively shuffled to calculate the growth rate μ and death rate θ in Equations 8 and 9. Equations 8 and 9 are based on mass balance principles and consist besides the growth and death rate, of the substrate feed $F_{in}$ , the specific viable cell to substrate yield $Y_{{sx}_{v}}$ and a specific net broth-mass change D accounting for feed addition, evaporation, and gas/liquid mass transfer contributions. The mechanistic model can be regarded as an extended version of previous mechanistic models addressing a similar industrial process, enabled by capacitance data (Albaek et al., 2011; Mears et al., 2017). Multiple biomass and substrate balance equations with a similar overall structure have been reported previously; therefore, the differential equations presented in Equations 8 and 9 are not considered novel in a general sense (Frame and Hu, 1991; Miller et al., 1988). Rather, they are used here as a standard unstructured mass-balance framework for viable cell growth and substrate consumption.

**TABLE 3:** Overview of the kinetic death and growth model candidates that have been selected for this study.

Name	Equation		References
Growth models $\left[\mu \right]$			
Monod-type	$\mu_{\max}\cdot \frac{S}{K_{s}+S}\cdot \frac{\text{DO}}{DO+K_{do}}$	(10)	(Bajpai and Reuss, 1980; Humphrey, 1979; Monod, 1949)
Contois-type	$\mu_{\max}\cdot \frac{S}{K_{s}\cdot X+S}\cdot \frac{\text{DO}}{DO+K_{do}}$	(11)	(Abdullah et al., 2016; Contois, 1959; Humphrey, 1979)
Moser-type	$\mu_{\max}\cdot \frac{S^{n}}{K_{s}+S^{n}}\cdot \frac{\text{DO}}{DO+K_{do}}$	(12)	(Ardestani and Kasebkar, 2014; Moser, 1958)
Tessier-type	$\mu_{\max}\cdot \left(1-e^{-\frac{s}{K_{s}}}\right)\cdot \frac{\text{DO}}{DO+K_{do}}$	(13)	(Humphrey, 1979)
Death models [ $\left.\theta \right]$			
Reversed Monod-type	$\theta_{\max}\cdot \frac{D_{S}}{D_{s}+S}\cdot \frac{K_{do_{death}}}{DO+K_{do_{death}}}$	(14)	(Amribt et al., 2013; Kyriakopoulos et al., 2018)
Autolysis	$\theta_{\max}$	(15)	(Bajpai and Reuss, 1980; Kyriakopoulos et al., 2018; Liu et al., 2016)
Craven-type	$\theta_{\max}\cdot \frac{\mu_{I}}{\mu +\mu_{I}}\cdot \frac{K_{do_{death}}}{DO+K_{do_{death}}}$	(16)	(Craven et al., 2012)
O’Brien-type	$\theta_{\max}\cdot \frac{\mu_{\max-}\;\mu }{\mu_{\max}}\cdot \frac{K_{do_{death}}}{DO+K_{do_{death}}}$	(17)	(O’Brien et al., 2021)

Originally, the growth and death models depended solely on substrate concentration but can easily be extended with additional inhibition terms (Muloiwa et al., 2020). Therefore, oxygen is also integrated as a secondary limiting factor in both growth and death kinetics. For the growth models, oxygen limitation is represented multiplicatively, such that the specific growth rate decreases when either substrate or oxygen becomes scarce.

$$\text{Viable cells }\left(X_{v}\right)\;\frac{dX_{v}}{\text{dt}}=\left(\mu -\theta \right)*X_{v}-DX_{v}$$ (8)

$$\text{Substrate }\left(S\right)\;\frac{\text{dS}}{\text{dt}}=\frac{F_{\text{in}}*C_{f}}{W}-\mu *\frac{X_{v}}{{Y_{\text{sx}}}_{v}}-\text{DS}$$ (9)

The selected growth models, namely, Monod, Contois, Moser, and Tessier (Equations 10–13) differ primarily in how they describe saturation behavior with respect to substrate availability, ranging from simple hyperbolic relationships to more flexible or exponential forms. The Contois model additionally accounts for biomass-dependent substrate limitation, which suggests reduced growth efficiency at higher cell densities (Contois, 1959; Monod, 1949)

Cell death is modeled in the same way, using rate expressions that increase under unfavorable conditions. The inverse models by Monod, Craven, and O’Brien (Equations 14, 16 and 17) describe death as a function of substrate and oxygen limitation, with some formulatons explicitly linking the death rate to the growth rate, thereby coupling growth inhibition and cell loss. For the Moser model the parameter $K_{S}$ must have units of $S^{n}$ . In contrast, the autolysis model (Equation 15) is independent of oxygen availability and represents cell death driven more by intrinsic degradation processes than by environmental constraints. Although oxygen availability may only become critical under severely depleted conditions, its inclusion in the kinetic expressions allows the model to capture the transition from growth to stress-induced death in a mechanistically consistent manner (Ardestani and Kasebkar, 2014; Muloiwa et al., 2020)

To mitigate the risk of entrapment in local minima, which is a common challenge in non-linear dynamic models, a Differential Evolution (DE) algorithm was employed as implemented in the SciPy library (Storn and Price, 1997). The optimization used the ‘best1bin’ strategy to balance convergence speed and population diversity. A population size multiplier of 6 was selected to ensure adequate coverage of the high-dimensional parameter space, and the evolutionary process was executed for a maximum of 30 generations. A relaxed tolerance of 0.01 was applied, allowing termination when improvements fell below 1%. To enhance accuracy without excessive computational cost, the algorithm employed the polish=True option, which performs a final local optimization using the L-BFGS-B method. This two-step approach combines global exploration with local refinement, enabling efficient parameter estimation while avoiding the computational burden of a high-accuracy global search alone (Moles et al., 2003; Storn and Price, 1997). All parameters were constrained within biologically plausible bounds (Supplementary Table S1, Supporting Information) to prevent non-physical solutions. Parameter estimation was performed by minimizing an objective function based on the sum of the normalized mean squared errors between simulated and experimental values for the pseudo-viable biomass and the substrate concentration in Fermentation 1 and Fermentation 2. Each individual normalized mean error ( $\left.NMSE\right)$ was calculated as shown in Equation 18:

$$NMSE=\frac{\frac{1}{n}\sum_{i=1}^{n}{\left(y_{i}-{\hat{y}}_{i}\right)}^{2}}{{\left(y_{\max}-y_{\min}\right)}^{2}}$$ (18)

where $y_{i}$ and ${\hat{y}}_{i}$ are the experimental and simulated values, respectively, and n is the number of observations for the respective data series.

In addition to the optimization objective, the Akaike Information Criterion (AIC) was used to evaluate model performance while accounting for differences in the number of fitted parameters among the growth and death model combinations (Akaike, 1974; Lovric, 2025). Since the online biomass signal contained substantially more observations than the offline substrate measurements, a balanced AIC was applied. In this approach, the four fitted data series were assigned equal weight by calculating the mean of the trajectory-specific normalized mean squared errors, thereby preventing the model ranking from being dominated by the high number of online biomass measurements. Because the online biomass-related signals were densely sampled time series, residuals were expected to be temporally autocorrelated. Therefore, an autocorrelation-corrected effective sample size was used in the AIC calculation.

$$AIC_{\text{bal},\text{eff}}=n_{\text{eff},\text{total}}*\ln\left(MSE_{\text{bal}}\right)+2k$$ (19)

The balanced autocorrelation-corrected AIC was calculated as shown in Equation 19, where $MSE_{\text{bal}}$ is the mean of the normalized mean squared errors across the four fitted data series, $n_{\text{eff},\text{total}}$ is the autocorrelation-corrected effective number of observations, and k is the number of fitted model parameters. The effective sample size was calculated using an AR(1)-type correction of the form $n_{\text{eff}}=n\left(1-\rho \right)/\left(1+\rho \right)$ . The autocorrelation parameter ρ was estimated separately for each fitted trajectory, as the lag-1 Pearson autocorrelation of the residuals between the measured values and the corresponding model predictions (Pyper and Peterman, 2011; Zięba, 2010). This was done only on the pseudo-viable cell data. The corrected AIC was used as a supportive ranking criterion rather than as formal evidence for a uniquely optimal kinetic model structure, particularly when the NRMSE values were similar. In such cases, the final selection should rely on case-specific scientific and engineering judgement, considering both model performance and biological model plausibility.

To assess whether the mechanistic growth/death models provided explanatory value beyond a simple empirical time-course, each biomass and substrate trajectory was fitted with a quadratic baseline, $\hat{y}\left(t\right)=a+bt+ct^{2}$ (Draper and Smith, 2014). This low-parameter null model captures general curvature without additional biological assumptions or choices such as spline knot placement. Because it cannot fully represent complex growth, death, or regrowth dynamics, the comparison only serves as a supportive plausibility check, not as formal evidence of superiority over all empirical alternatives.

Part three aims to identify the missing biological rates in the model, such as the oxygen uptake rate. The yields expressed in terms of viable cell concentrations correlate linearly with the experimentally measured net growth rate. Biologically, however, these yields should be related to the actual growth rate of viable cells. Since the capacitance measurements only reveal the net growth rate ( $\left.\approx \mu -\theta \right)$ , the viable growth rate μ estimated from the model was used to determine the dynamic yield relationships, as denoted in Equation 20. The dynamic yields were described for substrate, oxygen, carbon dioxide, ammonium, and product formation. The offset in Equation 20 should be interpreted as an empirical intercept of a growth-associated yield function, not as a classical maintenance term (Lemperle et al., 2026). Since the corresponding rates are formulated as $\mu *X_{v}/Y_{iX_{v}}\left(\mu \right)$ , they approach zero as μ→0 and therefore do in fact not represent non-growth-associated maintenance fluxes. Explicit maintenance-like uptake terms and non-growth-associated product formation terms were evaluated but did not improve the predictive performance for the present dataset. In the presented simulations, the estimated viable growth rate μ remained positive throughout the investigated fermentations, although the net viable biomass balance became negative in some phases when θ>μ . Thus, growth-associated fluxes were still active even during periods of decreasing viable biomass. Under these conditions, the available data could not reliably distinguish strictly growth-associated fluxes from weak additional non-growth-associated terms. Therefore, the growth-associated formulation was retained as a pragmatic simplification for the present dataset and should not be interpreted as a general mechanistic conclusion for filamentous fungal models. Parameter estimation was then performed using local optimization by minimizing the loss function for each state.

$${\;Y}_{iX_{v}}=Slope_{i}*\;\mu +offset_{i}$$ (20)

$$\frac{dP}{dt}=\mu *\frac{X_{v}}{{Y_{px}}_{v}}-DP$$ (21)

The underlying model structure for the product concentration is given in Equation 21.

$$OUR=\frac{\mu \;*X_{v}}{{\;Y}_{O_{2}X_{v}}}$$ (22)

Carbon evolution rate, oxygen uptake rate and ammonia uptake rate are described in the following way, given the OUR as an example structure in Equation 22.

To accurately model dissolved oxygen (DO) dynamics in the bioreactor, crucial for a full bioprocess model, it is necessary to describe both the oxygen uptake rate (OUR) and the oxygen transfer rate (OTR), the latter being defined as the product of the oxygen volumetric mass transfer coefficient ( $k_{L}a$ ) and the DO driving force. Given the significant influence of viscosity on oxygen transfer in industrially relevant fungal fermentation processes, this factor cannot be neglected (Garcia-Ochoa and Gomez, 2004). To account for this, a data-driven soft sensor was trained using data from Fermentation 1 and 2. In addition to accumulated values as well as rates of ammonia flow, CER, OUR, agitation and aeration, the bacterial mode capacitance measurement is integrated as an input feature of the ML model. The input features were scaled by the initial reactor weight to be scale-independent, which has been employed before in Lemperle et al. (2026).

A detailed comparison of 2 common time-dependent (attention-based Long Short-Term Memory (LSTM) and LSTM) and 2 time-independent (LGBM and ANN) machine learning models, which were investigated can be found in the supporting information (Gong et al., 2025; Nadda et al., 2024). Additionally, the leave-one-group-out (LOGO) and the k-fold cross validation method (kf) have been tested for each of those models. The final result is a hybrid bioprocess model that integrates the ML viscosity soft sensor into the set of differential equations, visualized in Figure 3. The resulting hybrid bioprocess model was subsequently evaluated with Fermentation 3 and 4.

**FIGURE 3:**
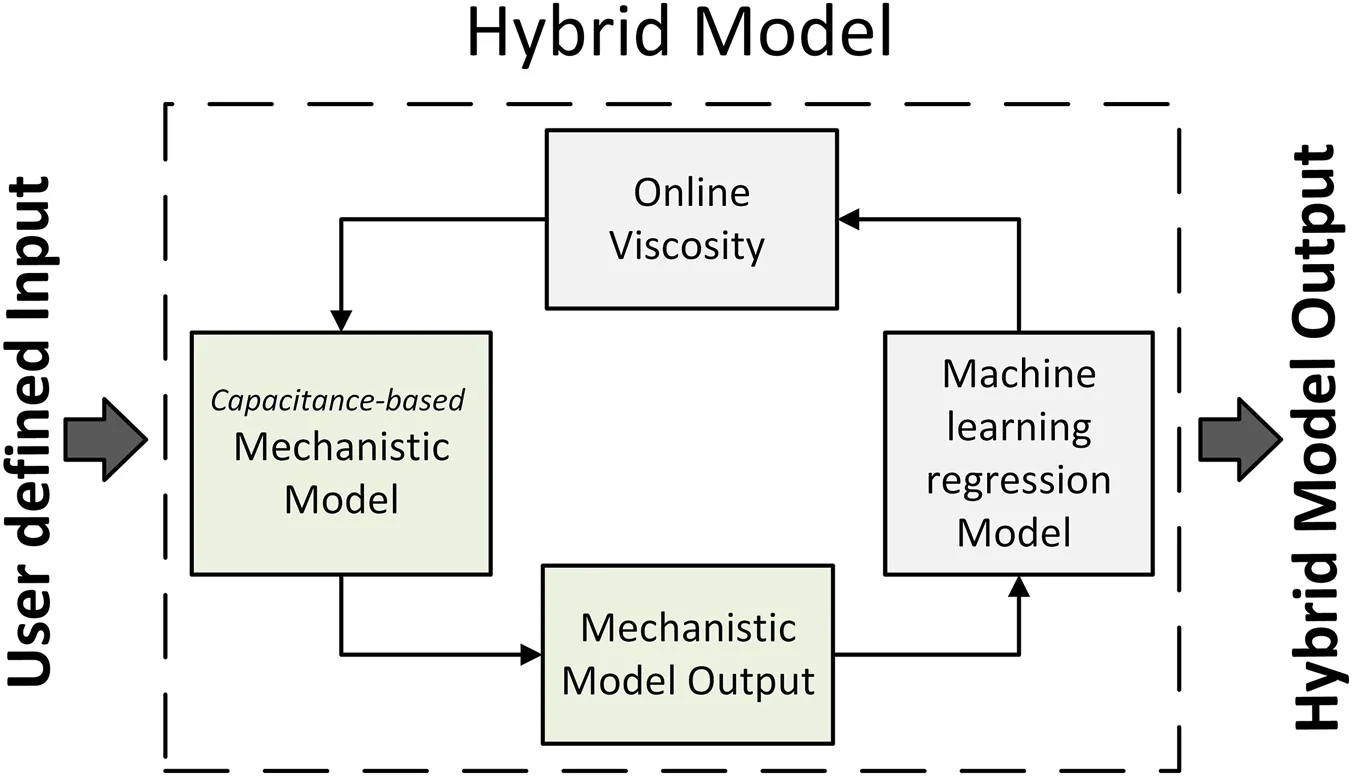
Schematic overview of the hybrid model that is constructed in this study. The ML model is integrated into the set of ordinary differential equations and solved iteratively. Further details of the ML models, including the input features, are presented and discussed in Figure 4.

**FIGURE 4:**
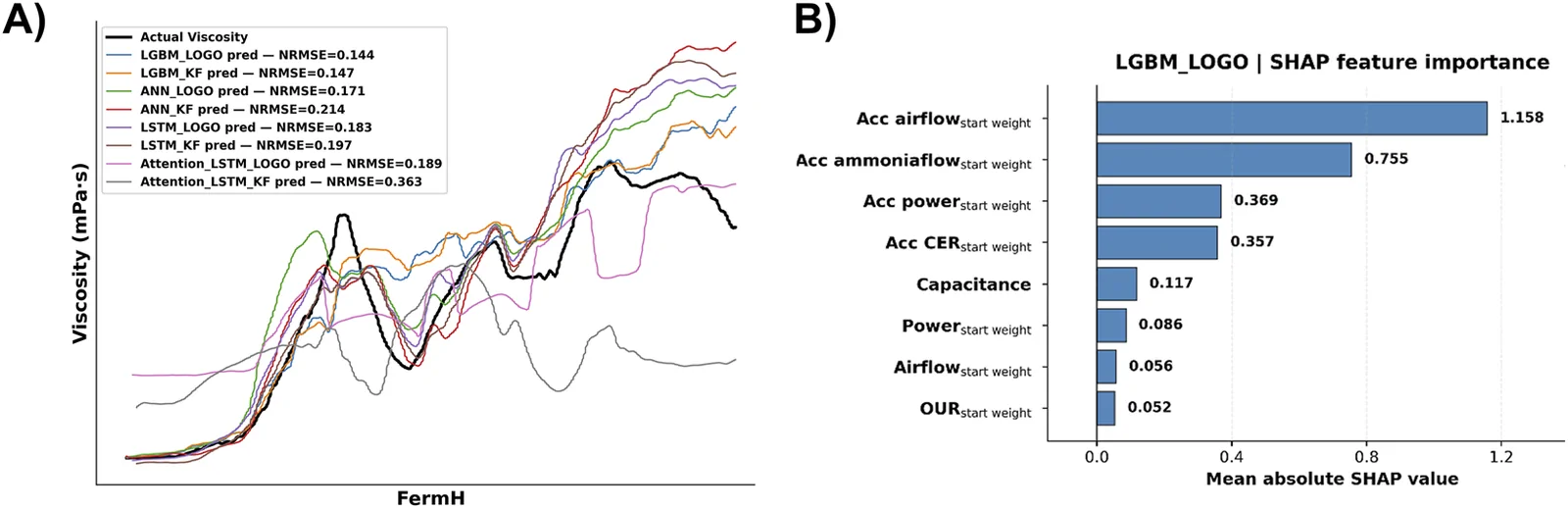
Validation results for fermentation 3 of the data-driven viscosity soft sensor. In this study, only two fermentation runs were used as training data. **(A)** Predicted and measured viscosity dynamics for Fermentation 3. **(B)** Feature importance analysis showing the impact of each input feature on the LGBM_LOGO model output.

For this proof-of-concept study, successful model performance was defined as a prediction accuracy for product concentration with an NRMSE below a 10% deviation, while deviations in the intermediate states, specifically flow rate and biomass, were used to determine the limits of the modeling framework. This is consistent with similar industry-relevant results reported in the literature, where prediction errors for the product range varied between 4.3% and 26.2%, depending on the process conditions (Albæk, 2012; Gernaey et al., 2010; Mears et al., 2017; Rydal et al., 2024).

## Results

### Fermentation data

Figure 5 presents normalized fermentation data for the three dual-frequency measurements, Cole-Cole α, critical frequency $f_{c}$ , feed rate, and DO with Cole-Cole α inherently valued between 0 and 1. Fermentations 1 to 3 (orange, red and blue trajectory) indicate significant viable cell growth, linked with dynamic growth and death phases, while Fermentation 3 (blue trajectory) reaches the highest total viable cell concentration. For Fermentation 2 (red trajectory), capacitance closely mirrored the feed rate throughout the first two growth phases ( $\mu_{1}=$ 0.07 h^-1^ and $\mu_{2}=$ 0.025 h^-1^). After the start of the final phase ( $\mu_{3}=$ 0.005 h^-1^), cell concentration declines for an extended period, indicating that cell death exceeds growth. Midway through this phase, the trend reversed, and growth once again surpassed cell death. Fermentation 4 (green trajectory), was carried out at a fixed feed rate that was significantly lower than in the other runs, resulting in a reduced concentration of viable cells. Accordingly, the observed differences in viable cell concentration reflect the different process conditions: Fermentations 1 and 3 had variable oxygen transfer rates (and thus variable feed rates), while fermentations 2 and 4 followed predefined feed strategies.

**FIGURE 5:**
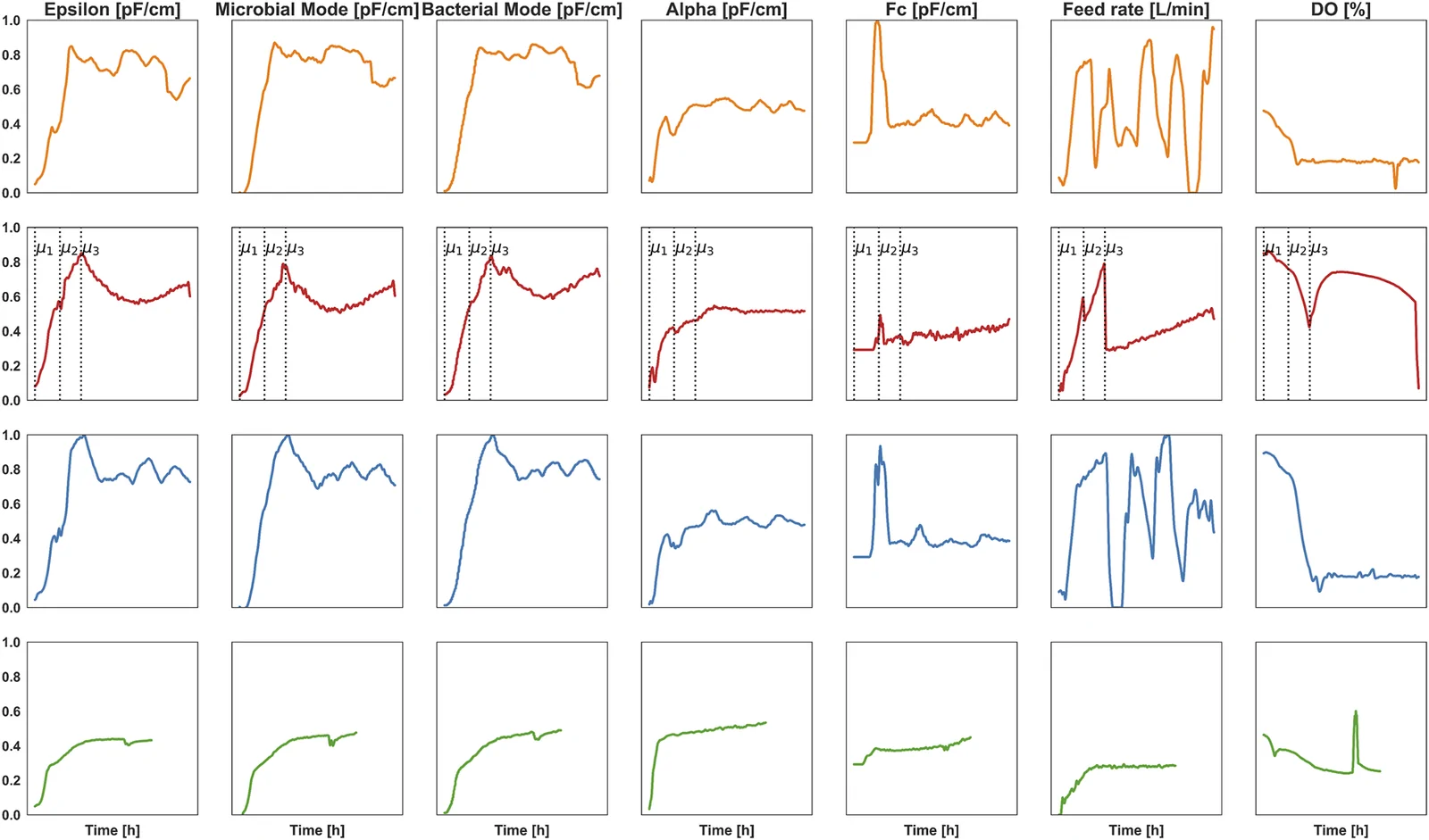
Overview of the most important capacitance probe measurements as well as feed rate and dissolved oxygen for all 4 fermentation runs. Orange: fermentation 1, red: fermentation 2, blue: fermentation 3 and green: fermentation 4, as denoted in Table 1.

During the outgrowth phase, Δε exhibits deviations compared to the microbial and bacterial modes. As demonstrated in Figure 5, the $f_{c}$ demonstrates a profound peak during the outgrowth phase of Fermentations 1, 3 and, to a lesser extent, 2, after which it decreases and remains relatively stable for the remainder of the process. This sudden rise in $f_{c}$ coincides with a decrease of the Cole-Cole α and a damping effect on Δε at that exact point of time. The only notable difference between bacterial and microbial mode occurs within the first few process hours, during which the microbial mode displays negative values, indicating measurement artifacts. The dynamics of Cole-Cole-α correlate well with the general trends of the dual-frequency modes and with the feed rate for each fermentation process.

The qualitative pre-modelling analysis shows that, due to the strong influence of $f_{c}$ on Δε and the measurement artifacts observed in the first process hours of the microbial mode, the bacterial mode seems to provide the most robust representation of the viable cell dynamics. Therefore, considering this data set, the bacterial mode is selected as the capacitance measurement that suits the development of the bioprocess model most.

## Model development

### Physical $k_{L}a$ correlation

Based on the modelling workflow, shown in Figure 2, the first step is to develop a suitable correlation model for the oxygen mass transfer. Figure 6 illustrates the intra-batch design together with the dynamics of agitation, aeration, and pressure during Fermentation 1. The results indicate that the new intra-batch design, which covers all corner points and repeatedly returns through the center point within a single fermentation, can be successfully implemented in a DO-controlled process, as seen in Figure 5.

**FIGURE 6:**
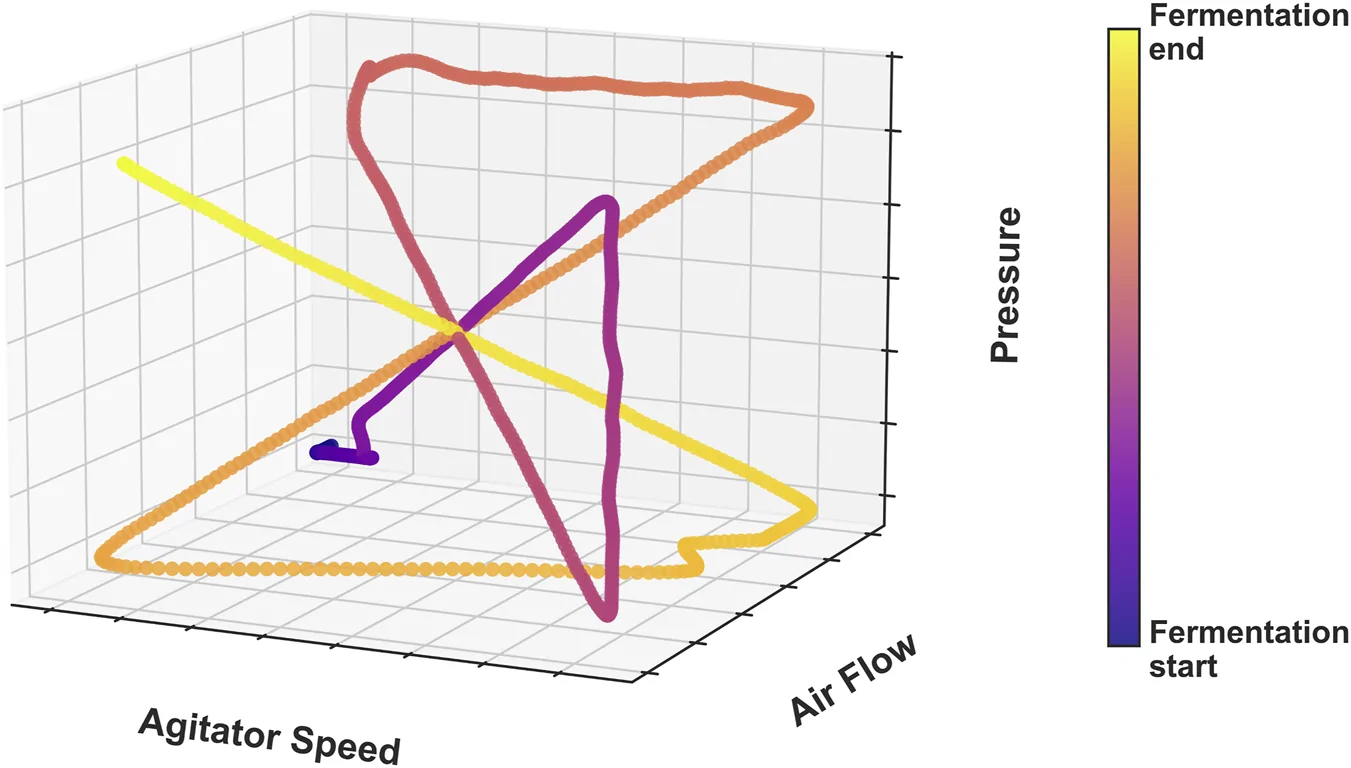
Intra-batch design, showing experimental agitation, airflow, and pressure dynamics over fermentation time, represented by a color gradient.

The results of the empirical $k_{L}a$ correlation are presented in Figure 7. The high coefficient of determination ( $R^{2}=0.95$ ) indicates strong agreement between the measured and fitted $k_{L}a$ values, although 339 of 950 data points (35.7%)fall outside the 30% deviation band, indicating some uncertainty in those estimates, which matches results with previously published $k_{L}a$ correlations (Albaek et al., 2011). Validation of the new correlation against experimental $k_{L}a$ data from fermentation 3 reveals that the model predicts the $k_{L}a$ dynamics with a relative error of 8%, which matches the accuracy achieved by the correlation reported in Lemperle et al. (2026) based on two fermentation runs. The estimated parameters ( C=7.34±0.84; a=0.55±0.02,b=0.5±0.03,c=−0.789±0.01 ) are in the expected range and except the parameter C, differ only slightly from the ones presented in Lemperle et al. (2026), C=11.348 ± 0.732,a=0.499 ± 0.007,b=0.484 ± 0.013,and c=−0.699 ± 0.007 (Lemperle et al., 2026).

**FIGURE 7:**
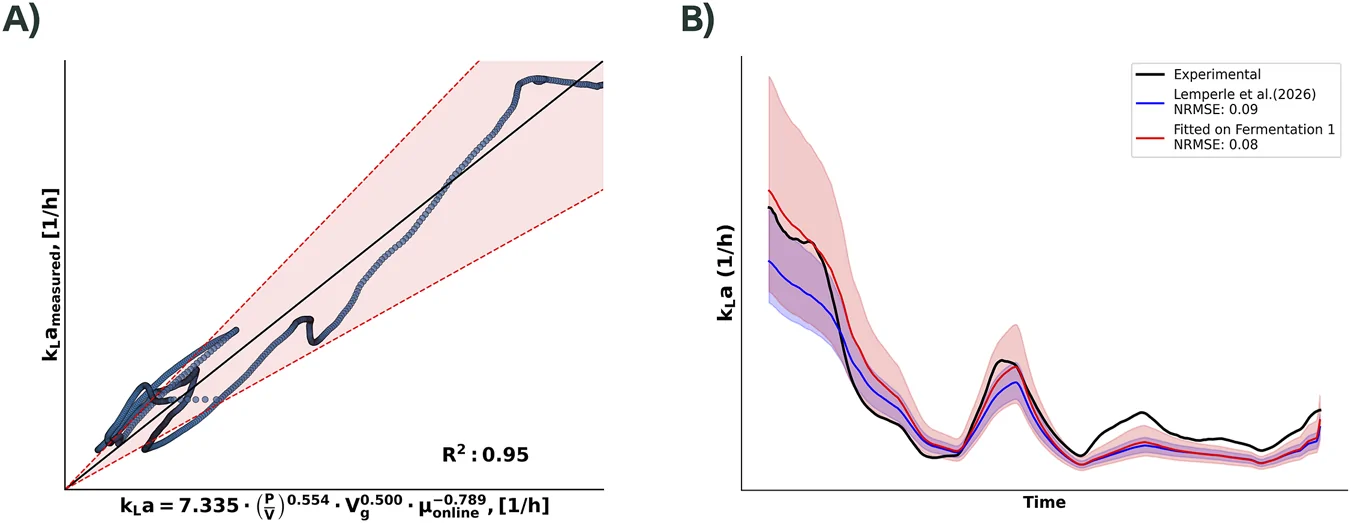
**(A)** Fitted $k_{L}a$ model using data from Fermentation 1 against the measured $k_{L}a$ , a line illustrating a perfect fit and a ± 30% error bounds area, shaded in red. **(B)** Experimental $k_{L}a$ of Fermentation 3 and the $k_{L}a$ model from Lemperle et al. (2026) as well as the one fitted on Fermentation 1 (Lemperle et al., 2026). Uncertainty bands were generated via Monte Carlo simulation ( n=1000 ), sampling model parameters from normal distributions based on their estimated means and standard deviations. Shaded regions indicate the 95% confidence interval (2.5^th^–97.5^th^ percentiles).

The uncertainty bands for the new $k_{L}a$ correlation indicate a greater uncertainty compared to the $k_{L}a$ prediction of Lemperle et al. (2026).

In the subsequent modelling phase, the online viscosity soft-sensor models were trained using Fermentations 1 and 2 and selected based on the lowest NRMSE on the external validation batch, Fermentation 3. The results in Figure 4 illustrate the performance of the different ML models on unseen data and indicate which models may be suitable to be integrated into the hybrid model. Since only a limited amount of training data and a single validation batch were available, these results should be interpreted as proof of concept rather than as definitive and final data-driven model development. A closer inspection reveals that, excluding the attention-based models, the LSTM, ANN, and LGBM models predict viscosity with NRMSE values ranging from 14.4% to 20.4%. These models captured the overall viscosity trend, although most miss the final details of the viscosity dynamics. Differences between training strategies, including leave-one-batch-out validation and k-fold cross-validation, were mainly observed for the ANN models, whereas the LSTM and LGBM models showed more similar performance across the two training methods. Given the limited number of batches available in the present study, the viscosity sub-model was selected based on a combination of predictive performance, robustness, and consistency with prior model behavior. The LGBM_LOGO model achieved a comparatively low NRMSE of 14.4% and was therefore selected as a suitable viscosity sub-model to be integrated into the hybrid model (Lemperle et al., 2026; Rydal et al., 2024). Figure 4B shows the importance of the input features based on SHAP values. As the model was trained on only two fermentations, these values should be interpreted only as an indication of the model’s behavior rather than as definitive mechanistic conclusions. Nevertheless, the accumulated input features were most important for this specific model, which is consistent with previous studies (Lemperle et al., 2026). Additional details of the ML model hyperparameters and training procedures can be found in the supporting information provided in Supplementary Tables S2 and S3.

#### Biological model

The first step of the biological model development is to determine the CDW-anchored one point calibration that converts the electrical output of the capacitance probe, expressed as $\frac{pF}{cm}$ , into a more practical unit, namely, the mass of viable cells per fermentation broth mass, e.g., $\frac{g}{kg}$ . Therefore, CDW data provide a reference point for calibrating the capacitance signal to a pseudo-viable cell concentration. In this example, applying a one-point calibration results in a calibration factor of 0.45, as visualized in Figure 8. The selected calibration point assumes that the viable fraction of the CDW is 1 at that specific point. As a result, the CDW value and the pseudo-viable cell concentration are identical at the calibration point (fifth datapoint). This relationship is apparent when comparing Figures 8A,B, where the calibrated pseudo-viable cell concentration intersects the corresponding CDW measurement. The maximum viable fraction of 1.0 in Figure 8B is therefore imposed by the normalization procedure and should not be interpreted as an independent measurement of viability. Due to practical analytical limitations, caused by the industrial scope of the study, no additional offline measurements could be used to further validate the assumptions.

**FIGURE 8:**
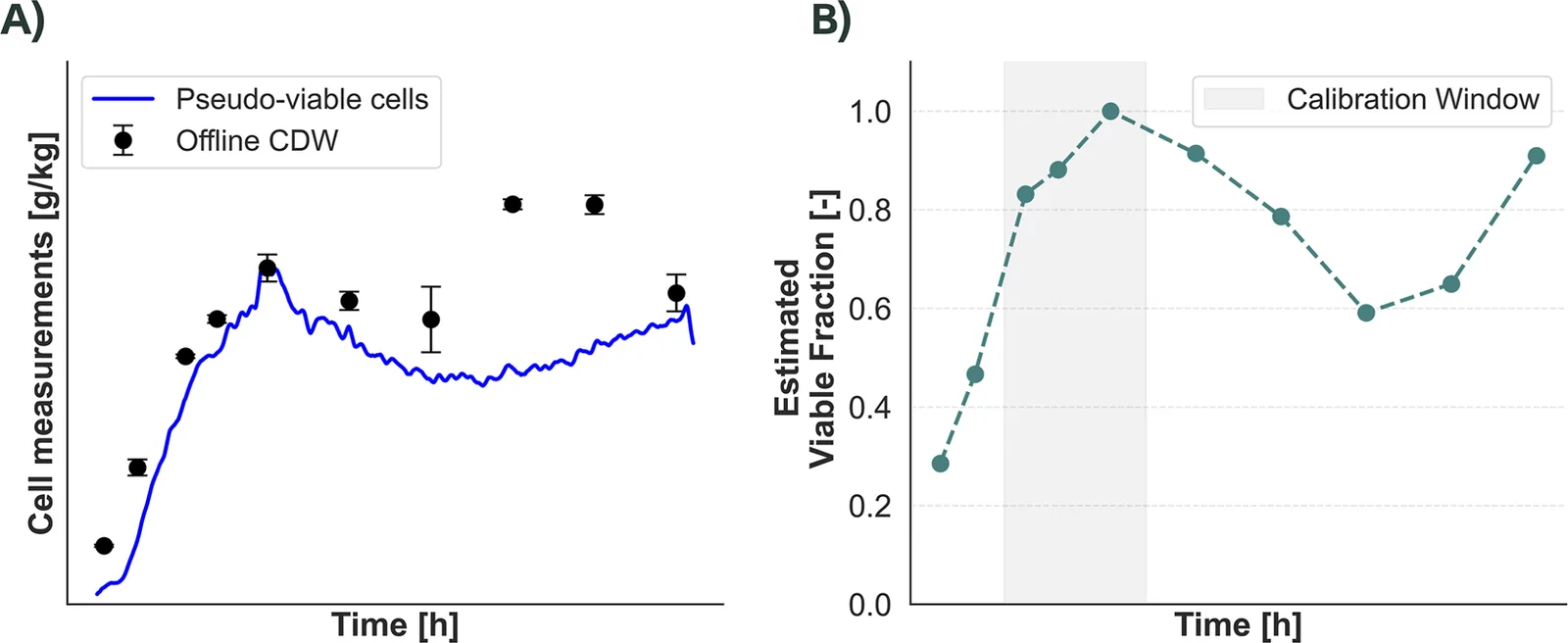
Visualization of the results of **(A)** the pseudo viable cell against the original CDW measurement over time **(B)** the viable cell fraction of the CDW over time. The peak value of $v_{f_{i}}=1$ results from the normalization used to define m and should not be interpreted as an independent viability measurement.

Following the calibration, the pseudo-viable cell concentration lies near the upper boundary of the CDW values during the outgrowth phase, as illustrated in Figure 8A. This is consistent with the interpretation that the pseudo-viable cell concentration represents only the viable-cell-associated fraction of the biomass, whereas the CDW measurement may additionally include residual material from dead cells and other insoluble solids. The identified factor can subsequently be used for all other batches as well and does not have to be reidentified for each batch or future work addressing this strain and fermentation process.

In the next stage of the modelling process, the selected growth and death model combinations were fitted and evaluated. Table 4 lists the combinations of models for Fermentation 1 and Fermentation 2 based on the average NRMSE, the balanced and autocorrelation corrected Akaike Information Criterion difference, ΔAIC , and the reduction in normalized residual sum of squares relative to the quadratic baseline.

**TABLE 4:** Top 10 combination of the growth and death models for Fermentation 1 and Fermentation 2. The autocorrelation factor ρ for the AIC of the pseudo-viable cell data is bound to the maximum value of 0.95 for all models.

Combination	Growth model	Death model	Average NRMSE [%] (objective function)	Δ AIC [-]	Number of parameter	$RSS_{reduction}$ [%]
1	Monod	O’Brien	7.6	0.0	8	65
2	Monod	Craven	7.62	0.4	8	64.7
3	Tessier	Autolysis	7.64	−1.2	7	64
4	Moser	Monod Reversed	7.71	5.0	9	62.6
5	Tessier	Monod Reversed	7.73	2.6	8	62.5
6	Tessier	O’Brien	7.76	3.3	8	62
7	Contois	Autolysis	7.79	1.5	7	61.5
8	Monod	Monod Reversed	7.87	8.9	8	61.2
9	Contois	Craven	7.87	5.1	8	60.6
10	Moser	Autolysis	7.95	7.5	8	58.9

The ΔAIC was calculated relative to the model combination with the lowest NRMSE value. The RSS reduction describes the percentage decrease in normalized residual error compared with the non-mechanistic quadratic time-trend baseline.

The results presented in Table 4 show that several growth and death model combinations can be fitted to represent the dynamics of the experimental data from Fermentations 1 and 2 with similar accuracy. This is given by the NRMSE values, which span only a narrow range from 7.60% to 7.95%. The ΔAIC values provide an additional ranking criterion by accounting for the number of fitted parameters; however, the differences between the best-performing combinations remain relatively small and should therefore not be interpreted as definitive evidence for a single best model structure.

The RSS reduction further indicates that all tested model combinations perform better than the quadratic baseline model. However, this is expected, since the quadratic baseline represents a relatively weak benchmark and cannot capture biphasic growth, death, or regrowth dynamics. Therefore, the RSS reduction mainly demonstrates improved descriptive capability compared with a simple empirical trend, rather than proving that one specific growth–death mechanism is uniquely identifiable.

The combination of the Monod growth model and the O’Brien death model was selected for the final hybrid model because it provided the lowest NRMSE while remaining consistent with the overall trends indicated by the additional evaluation criteria. However, the differences between several model combinations were small, and the available dataset does not allow a statistically definitive discrimination between alternative biological mechanisms. Therefore, this selection is interpreted as a pragmatic and biologically plausible model choice for demonstrating the proposed hybrid modeling workflow under limited industrial data availability, rather than as conclusive evidence for a specific growth–death mechanism.

These models were first used to fit the remaining yield parameters to the experimental data and were subsequently integrated into the final bioprocess model.

Due to confidentiality restrictions, the final calibrated kinetic parameters are not disclosed; however, the model structure, parameter bounds, and optimization procedure are provided to ensure methodological transparency. The objective function of the optimization procedure, the parameter bounds, and the parameter-space exploration are provided in the Supporting Information in Supplementary Figures S1,S2, and Supplementary Table S1.

Figure 9 presents the hybrid model predictions for Fermentations 3 and 4. Fermentations 1 and 2 were used to develop the biological and physical model components, while Fermentation 3 was additionally used to select the data-driven viscosity model. Therefore, Fermentation 3 provides a limited validation potential for the complete hybrid model, whereas Fermentation 4 represents an independent validation batch. The primary state variable, product concentration, is predicted with satisfactory accuracy for both batches, and overall, the simulated product prediction agrees well with the corresponding experimental trends.

**FIGURE 9:**
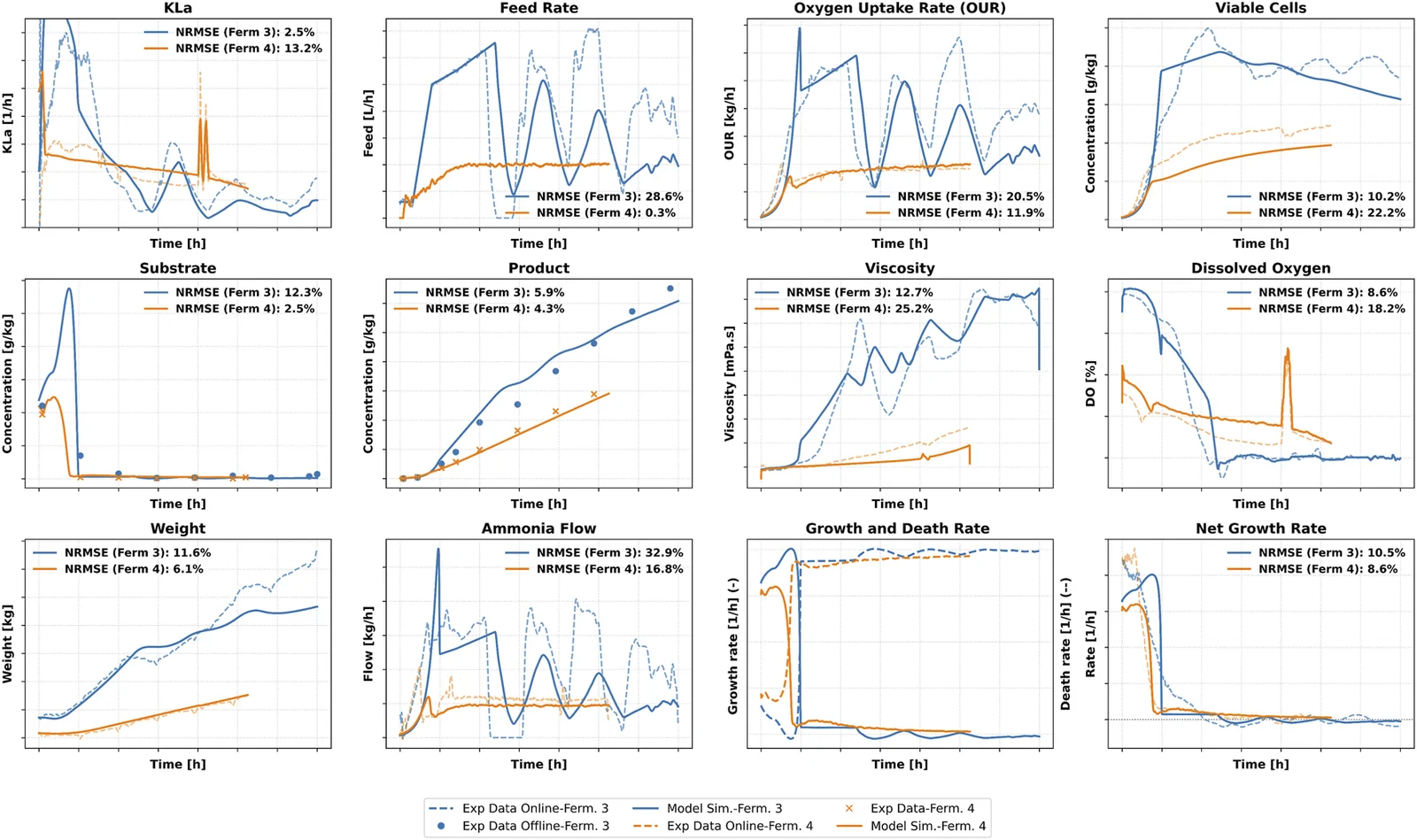
Full Hybrid bioprocess model simulation on Fermentation 3 (evaluation) and Fermentation 4 (validation): Closed loop for fermentation 3 and open loop simulation for Fermentation 4.

Although the model accurately predicts the OTR and OUR for a great part of Fermentation 3, the $k_{L}a$ is slightly underestimated in the later phase of the fermentation. As the model is very sensitive to a correct $k_{L}a$ and more general, OTR, this underprediction subsequently leads to an underestimation in most of the other states, such as feed rate, ammonia rate, weight, viable cell concentrations or OUR, explaining the observed discrepancies for Fermentation 3 (Garcia-Ochoa and Gomez, 2009). Fermentation 4 was simulated with open-loop settings, with a pre-defined feed rate and thus a better alignment between the experimental and predicted values can be observed compared to Fermentation 3.

The lower total prediction errors for the simulations of Fermentation 1 and Fermentation 2, which were used to develop the sub-models, presumably due to lower $k_{L}a$ inaccuracies, support this conclusion and underscore the high sensitivity of the model to correct $k_{L}a$ values. The results can be found in the supporting information Supplementary Figure S3.

#### Three-point robustness analysis with respect to m

To evaluate the robustness of the final simulation output to the calibration factor m , two additional calibration factors were derived from the one-point calibration value of 0.45. These correspond to a ±20% variation, yielding values of *m* = 0.36 and *m* = 0.54, respectively. For each calibration factor, the growth and death model as well as the dynamic yields were re-identified, and the simulations were repeated.

The resulting model performance, expressed as the normalized root mean squared error (NRMSE), is summarized in Supplementary Table S5 in the supporting Information. The deviations of the NRMSE from the base case are presented in Figure 10.

**FIGURE 10:**
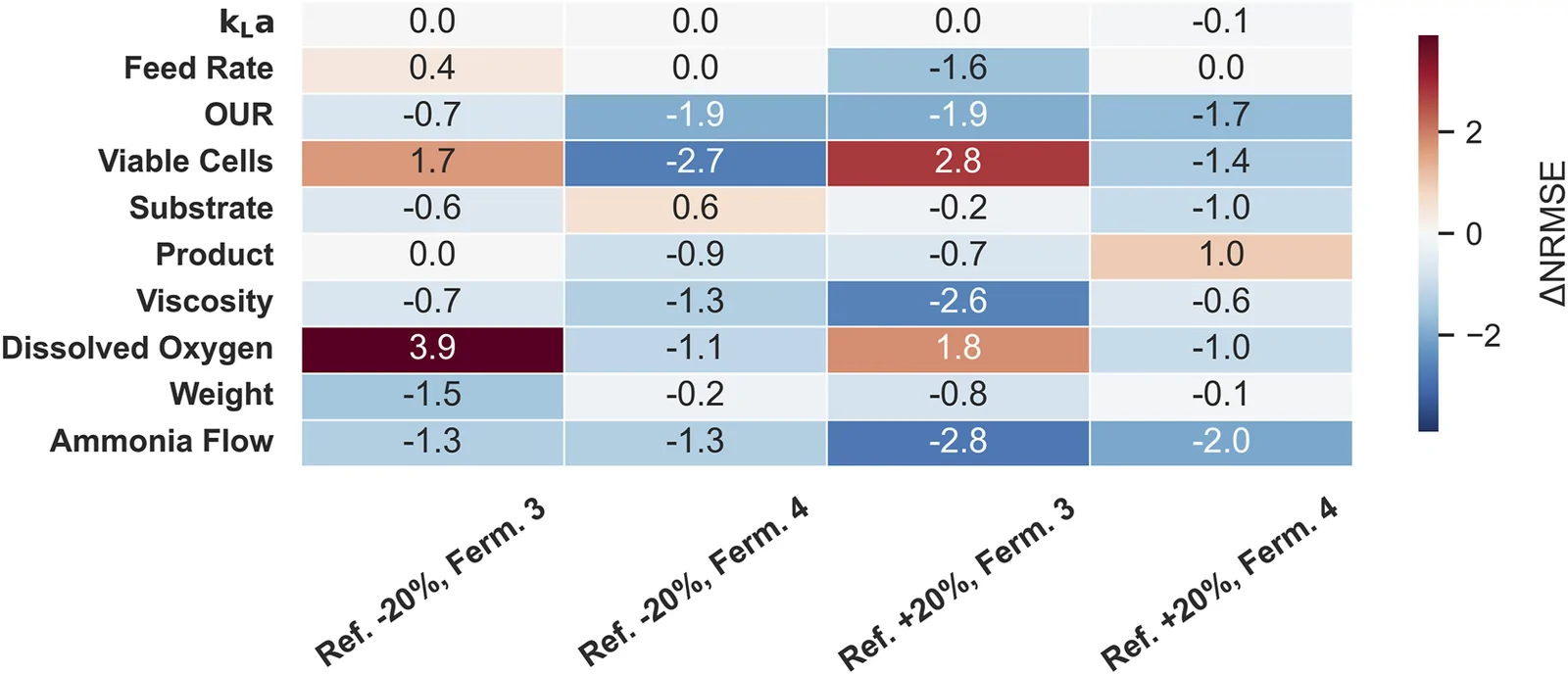
Deviation of the NRMSE relative to one-point calibration (m = 0.45), derived from the values given in Supplementary Table S5 (supporting information). Negative values indicate lower NRMSE than the baseline calibration; positive values indicate higher NRMSE.

Overall, the results reveal that variations in the calibration factor *m* within ±20% do not substantially affect model accuracy or the qualitative system dynamics. This observation is further supported by comparisons of the time series data presented in Supplementary Figures S4 and S5 in the supporting information. For most state variables, the prediction error remains below 3% across all calibration scenarios. Similar trends are observed for other key model states, like product or DO, suggesting that the model predictions are largely insensitive to a ±20% uncertainty of the calibration factor.

## Discussion

### Fermentation data

The five capacitance measurements obtained from four industrial pilot-plant fermentation runs provide comprehensive insights into viable fungal cells throughout an entire industrially relevant fermentation process. To the authors’ knowledge, such a level of detail has not yet been reported for industrial pilot plant fermentation runs.

The capacitance probe data of each process exhibits unique dynamics, which can be closely linked to underlying process conditions, primarily driven by nutrient availability in the bioreactor.

For instance, the observed peak in $f_{c}$ for Fermentation 1 and Fermentation 3 may provide information regarding the overall cell size. In the early fermentation phase, the environment is relatively rich in dissolved oxygen and supplemented with high substrate feed rates, conditions that seem to drive a high proliferation rate. Once the dissolved oxygen concentration declines toward its setpoint, the environmental conditions shift accordingly, indicated by the downwards shift of $f_{c}$ , causing this trend to reverse back to favor cell growth instead, leading to larger cells (Pires et al., 2024; Smith et al., 2015; Wobill et al., 2025).

Overall, the trend of the Cole-Cole α parameter suggests that, across all fermentations, the broth gradually shifts from a relatively homogeneous suspension towards a more heterogeneous culture during the process. However, despite substantial variations in process conditions, the changes indicated by α remain less pronounced than expected, which may reflect the robustness of the strain. Nevertheless, the results at low viable biomass concentrations should be interpreted with caution, where the quality of fit of the Cole–Cole model is typically poor.

Furthermore, only a comprehensive comparison of all three dual-capacitance measurements leads to the conclusion that the bacterial mode is the most robust signal to represent the viable biomass in the bioreactor for modelling purposes for this specific data set. The underlying reasons for the observations of measurement artifacts may provide valuable insights into the dielectric response of the culture and should be investigated in future work.

### Model development

The results of the intra-batch experimental design and calibration of the empirical $k_{L}a$ correlation further confirm that the intra-batch design can be used to generate a dataset to effectively calibrate an empirical $k_{L}a$ model, thereby reducing experimental efforts and costs compared to traditional approaches (Albaek et al., 2011).

The results also demonstrate that this approach can be further advanced to the point at which only online data of a single fermentation batch is required, thereby further reducing experimental effort. However, this comes at the cost of slightly increased uncertainty, and this trade-off must be evaluated on a case-by-case basis. Although the identifiability of parameters in using empirical power law correlations is a known problem, this limitation is inherent to the model itself and independent of the estimation method, thus it does not affect the suitability of the approach (Garcia-Ochoa et al., 2010).

The framework for constructing an online-data-based, viscosity-dependent $k_{L}a$ correlation combined with a data-driven viscosity sensor, as presented by Lemperle et al. (2026), is successfully applied in this study. Although only a limited number of data points were available for training the machine-learning models, namely, from two fermentation runs, Fermentation 1 and Fermentation 2, the proposed framework delivered reasonable results for most of the tested ML models. It is likely that additional data would further improve the machine learning model, or improve its generalizability if that is required, as presented in Lemperle et al. (2026), while being cautious of the known limitation associated with data-driven modelling (Lemperle et al., 2026; Mowbray et al., 2021; Oliveira, 2004). The results suggest that, even with limited data, a data-driven soft sensor for viscosity prediction can be developed at a proof-of-concept level.

All time-dependent machine learning models, apart from the attention-based LSTM, showed good predictive performance (Figure 4). This indicates that the temporal dependency of the fermentation process is an important source of information and should be considered on a case-by-case basis in future modelling work. However, the attention-based LSTM did not improve predictive performance compared with the standard LSTM. This might be expected, as attention-based models typically require larger datasets than were available in this study. This agrees with literature that reports the need for large data sets needed to train attention-based ML models (Lee et al., 2019; Mueed Hafiz et al., 2021). Furthermore, the results emphasize that the choice of hyperparameter optimization and validation strategy has an impact on some of the model’s performance, which aligns with similar reported observation from the literature (Hewamalage et al., 2021; Ke et al., 2017; Lim and Zohren, 2020; Probst et al., 2018). Further, calibrating the online capacitance data to a pseudo-viable cell concentration successfully provides an approximate viable biomass state that can be used for the development of the biological sub-models, such as growth or death models. While this provides a strong proof of concept, the calibration method relies on several assumptions rather than independent analytical confirmation. Future work should therefore focus on validating the assumptions towards cell viability, provided that additional analytical resources are available.

It is important to note that the modelling framework can deal with uncertainty of the CDW-based one-point calibration, which would be caused for instance by incorrect assumptions. This is demonstrated by a robustness analysis of the calibration factor m towards the model output accuracy. Two additional values of the calibration factor m were evaluated within the proposed workflow. A ±20% perturbation of m had only a minor effect on the overall model accuracy and dynamic behavior after parameter re-estimation, indicating that the integrated model output is relatively insensitive to moderate uncertainty in the CDW-derived calibration anchor. However, because m primarily rescales the inferred pseudo-viable biomass state, part of this robustness can be attributed to structural compensation by biomass-related yield parameters, such as $Y_{P/X_{v}}$ or $Y_{O_{2}/X_{v}}$ . Thus, the analysis supports robustness of only the final model predictions within the tested perturbation range but does not independently validate the absolute viable biomass scale. Still, this is a positive and important finding, as it indicates that the framework can tolerate some uncertainty in m, the only calibration step that requires at least one offline CDW measurement.

As a high level of precision in the biological model parameters is not always required in industrial practice, where key variables such as product concentration are of primary importance, the present proof-of-concept demonstrates that the workflow can support model development using only a single CDW anchor point and without the need for offline rheology measurements.

The development of the hybrid model described here now spans three batches and requires only one CDW measurement, taken at a process point where the viable-cell fraction of the CDW can reasonably be assumed to be 1 under ideal conditions. This represents a reduction in required fermentation runs of at least 66.7% (from 9 to 3 fermentation runs) compared with previous modelling work on a similar process by for instance, Albæk, (2012). In addition, almost no further offline sampling is required apart from this single CDW calibration point, which substantially reduces laboratory labor. Viscosity and CDW offline measurements can easily take between 1 and 2 h per four fermentation runs; over multiple days and with 10–15 data points per fermentation, the approach presented here leads to a considerable reduction in manual work. It translates to a reduction of 90%–95% for time-consuming and lengthy CDW measurements. Those key benefits are achieved by a modelling workflow, which avoids complex calibration procedures that are typically required for capacitance-based estimates of viable cell concentration (Dabros et al., 2009; Fehrenbach et al., 1992; Konakovsky et al., 2015; Luttmann et al., 2012; Rathore et al., 2021).

The parameter estimation workflow identified several growth and death model combinations with comparable NRMSE values, AIC and baseline reduction. The balanced Akaike Information Criterion was largely consistent with the NRMSE-based ranking, suggesting that the selected model was not solely favored by increased parameter complexity. Furthermore, the mechanistic models outperformed the non-mechanistic empirical baseline, supporting the relevance for describing the observed process dynamics with the presented growth and death models. While the relatively low NRMSE can be considered a successful outcome, the growth and death model also indicate a well-known practical parameter identifiability limitation (Raue et al., 2009). In total, 7–9 parameters were fitted using only the pseudo viable-cell concentration and limited offline substrate data, which represents an inherent limitation when fitting biological growth and death models under restricted data availability. In addition, when only viable biomass or capacitance-derived biomass is observed, the model primarily informs the net rate $\mu -k_{d}$ (Bellman and Åström, 1970). Separating this into an individual growth rate μ and death rate $k_{d}$ is therefore weakly identifiable. For academic model validation, independent information on dead cells would be valuable to improve the identifiability of growth and death contributions (Newton et al., 2017). However, adding such measurements would also compromise the pragmatic workflow proposed here, which aims to minimize additional offline sampling under industrial data limitations.

Future work could further investigate the robustness of the parameter optimization procedure, for example, through alternative weighting strategies, cross-validation, or uncertainty analysis.

Mass balances, particularly the carbon balance, may provide an additional plausibility check during this step, provided that the process is well characterized and known to exhibit reliable balance closure (Doran, 1995; Villadsen et al., 2011). Once the calibration has been performed, the resulting biomass estimate could be evaluated against the overall carbon balance. A pronounced mismatch may indicate that the conversion is not consistent with the available process data. However, such an assessment requires that other carbon-containing inputs and outputs are sufficiently well quantified (Villadsen et al., 2011).

The evaluation of the open- and closed-loop fermentations reveals that this framework is able to predict the fermentation trajectory, also for fermentations operated differently from the one used for model calibration. Although the product concentration is predicted with high accuracy in both cases, further improvements could be achieved by refining the physical components of the model, especially the $k_{L}a$ and the data-driven viscosity model.

From an industrial perspective, the continued generation of process data could enable further improvement of the data-driven component. Newly available data may be incorporated either by retraining the model on the combined historical and new data set or, depending on the model type, by updating an existing calibration through incremental or transfer-learning approaches (Chen et al., 2025; Hutter et al., 2021). However, such improvements still require validation, preprocessing, and assessment of model performance. The $k_{L}a$ correlation itself may only be improved by integrating advanced theoretical models rather than relying on an empirical correlation used in this study. However, the cost–benefit of such integration must be evaluated on a case-by-case basis, and to date, no example is known in which such an approach has proven beneficial for this type of application.

## Conclusion and outlook

The work presented in this study aims to reduce the need for laborious and extensive offline sampling, particularly cell dry weight (CDW) measurements as well as the number of fermentations required for the development of industrial bioprocess models. By incorporating online capacitance measurements, the proposed modelling workflow enables the use of real-time biomass-related information and reduces reliance on time-consuming offline analytics. The framework limits the number of required fermentations to three, while still ensuring full coverage of the design space for the empirical $k_{L}a$ correlation. In addition, it substantially reduces the need for offline CDW and rheology measurements. Together, these aspects contribute to a more efficient and streamlined model development workflow.

In this way, the study supports the broader objective of moving toward automated model development, which is increasingly targeted by the bioprocessing industry. The authors acknowledge that the proposed framework does not aim to achieve the highest level of biological rigor typically pursued in academic bioprocess modeling. This is mostly apparent in the modelling of the biological cell dynamics, which is caused by the large number of parameters for the growth and death models and the limited data about dead cells or substrate. Therefore, the final outcome should not be regarded as a definitive or fully verified model, but rather as a proof of concept for the proposed modelling framework. In this sense, it supports the transition toward Industry 4.0-oriented bioprocessing by presenting a novel hybrid modelling workflow that integrates capacitance data without requiring extensive capacitance calibration.

## Data Availability

The data and code underlying this article cannot be made publicly available due to confidentiality obligations associated with the industrial collaboration and the use of proprietary process data. Relevant methodological details, model structures, and parameterization procedures are described in the manuscript to support reproducibility within these constraints.

## Glossary

${Y_{SX}}_{v}$: Yield coefficient of viable biomass per substrate $\left(\frac{gX_{v}\;}{gS}\right)$
${Y_{O_{2}X}}_{v}$: Yield coefficient of viable biomass per $O_{2}\;\left(\frac{gX_{v}}{mol\;O_{2}}\right)$
${Y_{CO_{2}X}}_{v}$: Yield coefficient of viable biomass per $CO_{2}\;\left(\frac{gX_{v}}{mol\;CO_{2}}\right)$
$Y_{{\text{NH}}_{4}^{+}X_{v}}$: Yield coefficient of viable biomass per ${\text{NH}}_{4}^{+}\;\left(\frac{gX_{v}}{mol\;{\text{NH}}_{4}^{+}}\right)$
$Y_{PX_{v}}$: Yield coefficient of viable biomass per $\text{Product }\left(\frac{gX_{v}}{gP}\right)$
$F_{in}$: Substrate Feed $\left(\frac{kg}{h}\right)$
$X_{v}$: Pseudo viable cell concentration $\left(\frac{gX_{v}}{kg_{broth}}\right)$
S: Substrate concentration $\left(\frac{gS}{kg_{broth}}\right)$
DO: Dissolved Oxygen concentration $\left(\frac{mol\;DO}{kg_{broth}}\right)$
P: Product concentration $\left(\frac{gP}{kg_{broth}}\right)$
W: Weight broth $\left(kg_{broth}\right)$
$c_{F}$: Concentration feed $\left(\frac{g_{substrate}}{kg_{feed}}\right)$
$\rho_{b}$: Density broth ( $\frac{\text{kg}}{L}$ )
$V_{l}$: Volume of the liquid $\left(m^{3}\right)$
ΔCap: Capacitance measurement $\left(\frac{pF}{cm^{2}}\right)$
Δε: Delta epsilon $\left(\frac{pF}{cm^{2}}\right)$
$\Delta \varepsilon_{\text{microbial}}$: Microbial mode $\left(\frac{pF}{cm^{2}}\right)$
$\Delta \varepsilon_{\text{bacterial}}$: bacterial mode $\left(\frac{pF}{cm^{2}}\right)$
CDW: Cell dry weight $\left(\frac{gCDW}{kg}\right)$
$v_{f_{i}}$: Viable fraction of a specific CDW sample i $\left(-\right)$
μ: Growth rate (1h)
$\mu_{I}$: Intrinsic growth rate $\left(\frac{1}{h}\right)$
θ: Death rate $\left(\frac{1}{h}\right)$
$\theta_{\max}$: Maximum death rate $\left(\frac{1}{h}\right)$
$\mu_{\max}$: Maximum growth rate $\left(\frac{1}{h}\right)$
$K_{s}$: Substrate saturation constant for the growth rate $\left(\frac{gS}{kg_{broth}}\right)$
$D_{S}$: Substrate inhibition constant for the death rate $\left(\frac{gS}{kg_{broth}}\right)$
$K_{do}$: DO inhibition constant for the growth rate $\left(\frac{DO\left[\%\right]}{kg_{broth}}\right)$
$K_{do_{death}}$: DO inhibition constant for the death rate $\left(\frac{DO\left[\%\right]}{kg_{broth}}\right)$
$Slope_{i}$: Linear correlation factor for the dynamic yield $\left(\frac{gX_{v}*h\;}{g}\right)$
$offset_{i}$: Empirical intercept of the dynamic yield relation $\left(\frac{gX_{v}\;}{g}\right)$
m: Calibration factor for the capacitance-based biomass volume $\left(\frac{g_{cell}\;cm^{2}}{kg_{broth}\;pF}\right)$
