# Supplementary material for: Online capacitance-integrated development of a hybrid bioprocess model for industrial fungal fermentations
Source: Front Bioeng Biotechnol. 2026 Oct 5;14:1919748. doi: 10.3389/fbioe.2026.1919748 (PMC13677858; doi:10.3389/fbioe.2026.1919748)
Supplement: Supplementary file 1 [file Supplementaryfile1.docx]

# Supporting Information:

### Parameter space for growth and death models

Figure S1: Parameter space for the parameter used and estimated in the growth and death model optimization

Loss function

Figure S2: Loss function for the objective function for the best growth and death model. Until approx. 1750 simulation, the global simulation runs and, in the end, the local continues until the convergence criteria is meet.

### Parameter space for growth and death models

Table S1: Parameter space for the growth and death model candidate search

| **Parameter** | **Bounds (min–max)** |
| --- | --- |
| $\mu_{max}$ | 0.01 – 1.2 |
| $K_{s}$ | 0.001 – 30 |
| $\theta_{max}$ | 0.001 – 0.1 |
| $D_{s}$ | 0.001 – 30 |
| $Y_{S{X_{v}}_{slope}}$ | 0.01 – 10.0 |
| $Y_{S{X_{v}}_{offset}}$ | 0.001 – 5.0 |
| $n$ | 0.1 – 5.0 |
| $dK_{do}$ | 0.01 – 100.0 |
| $K_{do_{death}}$ | 1 – 20000.0 |

### Final bioprocess model

| Broth mass | $\frac{dW}{dt}= F_{in_{feed}}+OUR+$ *AUR*$- F_{{out}_{evap}}-CER$ |
| --- | --- |
| Pseudo viable cell | $\frac{dX_{v}}{dt}=\left( \mu-\theta\right)*X_{v} -DX_{v}$ |
| Substrate | $\frac{dS}{dt}=\frac{F_{in}*C_{f}}{W}-\mu*\frac{X_{v}}{{Y_{sx}}_{v}}- DS$ |
| Dissolved oxygen | $\frac{dDO}{dt}=K_{L}a*\left( DO^{*}-DO \right)-\mu*\frac{X}{Y_{O2X_{v}}}-D*DO$ |
| Product | $\frac{dP}{dt}=\mu*\frac{X_{v}}{{Y_{px}}_{v}} -DP$ |
| OUR | $OUR=\frac{\mu*X}{Y_{O_{2}X_{v}}}$│$CER=\frac{\mu*X}{Y_{{CO}_{2}X_{v}}}$ │$AUR=\frac{\mu*X}{Y_{\mathrm{NH}_{4}^{+}X_{v}}}$ |
| Yields | ${Y_{SX_{v}}, Y}_{O_{2}X_{v}},Y_{CO_{2}X_{v}},Y_{\mathrm{NH}_{4}^{+}X_{v}}, Y_{PX_{v}} =f(\mu$) |

### Viscosity data-driven sensor

Table S2: Hyperparameter values of the used ANN model as well as the training hyperparameter ranges for all ML models

| **Parameter** | **ANN Range (min)** | **ANN Range (max)** | | **ANN Actual Value** | **LSTM Range (min)** | **LSTM Range (max)** | **Attention LSTM Range (min)** | **Attention LSTM Range (max)** | **LGBM Range (min)** | **LGBM Range (max)** |
| --- | --- | --- | --- | --- | --- | --- | --- | --- | --- | --- |
| **Learning rate** | 0,0001 | 0,01 | 0,0003 | | 0,0001 | 0,01 | 0,0001 | 0,01 | 0,01 | 0,1 |
| **N neurons** | 16 | 128 | 85 | | 32 | 128 | 32 | 128 | N/A | N/A |
| **N of hidden layers** | 1 | 2 | 2 | | 1 | 2 | 1 | 2 | N/A | N/A |
| **Dropout** | 0 | 0,4 | 0,388 | | 0,1 | 0,5 | 0,1 | 0,5 | N/A | N/A |
| **l2** | 0 | 0,05 | 0,041 | | N/A | N/A | N/A | N/A | N/A | N/A |
| **Batch size** | 10 | 30 | 24 | | 32 | 128 | 32 | 128 | N/A | N/A |
| **Epochs** | 20 | 100 | 95 | | 20 | 50 | 20 | 50 | N/A | N/A |
| **Time steps** | N/A | N/A | N/A | | 5 | 40 | 5 | 40 | N/A | N/A |
| **N estimators** | N/A | N/A | N/A | | N/A | N/A | N/A | N/A | 50 | 1000 |
| **Max depth** | N/A | N/A | N/A | | N/A | N/A | N/A | N/A | 5 | 12 |
| **Min child samples** | N/A | N/A | N/A | | N/A | N/A | N/A | N/A | 0 | 10 |
| **Subsample** | N/A | N/A | N/A | | N/A | N/A | N/A | N/A | 0,6 | 1 |
| **Colsample bytree** | N/A | N/A | N/A | | N/A | N/A | N/A | N/A | 0,6 | 1 |
| **Reg alpha** | N/A | N/A | N/A | | N/A | N/A | N/A | N/A | 0 | 0,5 |
| **Reg lambda** | N/A | N/A | N/A | | N/A | N/A | N/A | N/A | 0 | 0,5 |

Table S3: Summary between both validation methods during the ML model training procedure

| **Description** | **LOGO** | **K-Fold (K=5)** |
| --- | --- | --- |
| **Method Name** | Leave-One-Group-Out | K-Fold Cross Validation |
| **Number of Splits/Folds** | 2 splits (1 per batch) | 5 splits (fixed) |
| **Split Strategy** | Leave entire batch out | Random stratified splits |
| **Validation Data per Fold** | 1 complete batch | 20% of all samples |
| **Training Data per Fold** | All other batches | 80% of all samples |
| **How Splits are Made** | Split by batch ID groups | Random sample splitting |

### Model Simulation for the processes used for model development:

Figure S3: Representing the closed (Fermentation 1) and open loop (Fermentation 2) simulations with the experimental data for the most crucial fermentation states.

Table S4: NRMSE of the final model simulation with the one-point calibration based on CDW as well as the reference$\pm$20% calibration factors

|  | **One-point calibration factor, based on CDW (**$\boldsymbol{m}\mathbf{= 0.45}$**)** | | **Reference-20% (**$\boldsymbol{m}\mathbf{= 0.3}\boldsymbol{6}$**)** | | **Reference+20% (**$\boldsymbol{m}\mathbf{= 0.5}\boldsymbol{4}$**)** | |
| --- | --- | --- | --- | --- | --- | --- |
| **Fermentation** | Ferm. 3 | Ferm. 4 | Ferm. 3 | Ferm. 4 | Ferm. 3 | Ferm. 4 |
|  | NRMSE [%] | | | | | |
| $\boldsymbol{k}_{\boldsymbol{L}}\boldsymbol{a}$ | 2.5 | 13 | 2.5 | 13 | 2.5 | 13 |
| **Feed Rate** | 28.3 | 0.3 | 29 | 0.3 | 28 | 0.3 |
| **OUR** | 20.3 | 11.9 | 19.8 | 10 | 19.7 | 10.4 |
| **Viable Cells** | 10.5 | 22.2 | 13.1 | 19.3 | 14.3 | 20 |
| **Substrate** | 12.3 | 2.6 | 11.7 | 3.3 | 12.1 | 1.6 |
| **Product** | 5.8 | 4.3 | 6 | 3.4 | 5.6 | 5.2 |
| **Viscosity** | 12.1 | 13.3 | 14.8 | 14 | 14 | 14.2 |
| **Dissolved Oxygen** | 11.7 | 20 | 13.1 | 20.4 | 12.3 | 20.6 |
| **Weight** | 12.8 | 6.1 | 13.1 | 5.9 | 12.8 | 6 |
| **Ammonia Flow** | 32.9 | 16.9 | 31.9 | 15.5 | 31.1 | 14.8 |

### Model Simulation for the evaluation data with Ref.-20 and Ref.+20%

Figure S4: Model Simulation for the evaluation data **with Ref.-20%.**

Figure S5: Model Simulation for the evaluation data **with Ref.+20%**
